# Biomimetic Wearable Sensors: Emerging Combination of Intelligence and Electronics

**DOI:** 10.1002/advs.202303264

**Published:** 2023-12-03

**Authors:** Donglei Pan, Jiawang Hu, Bin Wang, Xuanjie Xia, Yifan Cheng, Cheng‐Hua Wang, Yuan Lu

**Affiliations:** ^1^ College of Light Industry and Food Engineering Guangxi University Nanning Guangxi 530004 China; ^2^ Key Laboratory of Industrial Biocatalysis Ministry of Education Department of Chemical Engineering Tsinghua University Beijing 100084 China

**Keywords:** artificial intelligence, artificial sensory systems, biomimetic functional materials, synthetic biology, wearable sensing systems

## Abstract

Owing to the advancement of interdisciplinary concepts, for example, wearable electronics, bioelectronics, and intelligent sensing, during the microelectronics industrial revolution, nowadays, extensively mature wearable sensing devices have become new favorites in the noninvasive human healthcare industry. The combination of wearable sensing devices with bionics is driving frontier developments in various fields, such as personalized medical monitoring and flexible electronics, due to the superior biocompatibilities and diverse sensing mechanisms. It is noticed that the integration of desired functions into wearable device materials can be realized by grafting biomimetic intelligence. Therefore, herein, the mechanism by which biomimetic materials satisfy and further enhance system functionality is reviewed. Next, wearable artificial sensory systems that integrate biomimetic sensing into portable sensing devices are introduced, which have received significant attention from the industry owing to their novel sensing approaches and portabilities. To address the limitations encountered by important signal and data units in biomimetic wearable sensing systems, two paths forward are identified and current challenges and opportunities are presented in this field. In summary, this review provides a further comprehensive understanding of the development of biomimetic wearable sensing devices from both breadth and depth perspectives, offering valuable guidance for future research and application expansion of these devices.

## Graphical abstract

Wearable sensors are advanced noninvasive diagnostic tools for disease and health monitoring. By mimicking biological sensing strategies, sensors have achieved significant improvements in material functionality and detection mechanisms. This article summarizes the outstanding advantages of biomimetic wearable sensors in terms of material functionality and proposes reasonable recommendations to promote higher‐level sensing capabilities in multiple fields.

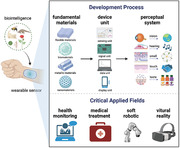

## 1 Introduction

Wearable sensing systems are a type of sensing devices that noninvasively or minimally invasively capture biochemical/mechanical signals of the human body and perform data analysis. They can conveniently and continuously monitor real‐time physiological signals related to the body,^[^ ^1^ ^]^ realizing human health monitoring^[^ ^2^ ^]^ and noninvasive personalized medicine.^[^ ^3^ ^]^ The international market for wearable medical sensing devices is expected to grow from $20.1 billion in 2021 to $83.9 billion in 2026,^[^ ^2c^ ^]^ indicating the promise of these devices to become exciting new‐generation portable remote medical devices. Additionally, wearable sensing systems can be used as skin sensory inputs of virtual reality (VR)/augmented reality (AR) systems, transmitting information, including temperature, pressure, and strain, to users for application in the human–machine interface (HMI).^[^ ^4^ ^]^ Currently, wearable sensing systems coupled with external signal‐conversion, control, and execution modules are also widely used in smart prosthetics^[^ ^5^ ^]^ and soft robotics.^[^ ^6^ ^]^

Compared to the case of previous generation of rigidly fixed sensors, research in the field of wearable sensors involves numerous scientific challenges, for instance, capture of bioelectronic interface signals, electronic transport in a flexible medium, and multimodal signal processing. Despite the development of wearable sensors, some pressing scientific questions, namely intelligent material response mechanisms, appropriate expression of material functionality, and breakthroughs in artificial perceptual dimensions, still need to be addressed.^[^ ^1^ ^]^ In this regard, the utilization of biomimetic technology is important. Organisms exhibit adaptive changes in response to external stimuli. They have accumulated unimaginable wisdom over billions of years. Therefore, investigation of the structural characteristics and stimulus‐response mechanisms of living organisms exhibits potential and high expectations for solving the aforementioned problems. Currently, the contributions of biomimetic technology to the development of wearable sensors are prominently demonstrated in terms of the functional capabilities of materials and sensor transmission.

Generally, a wearable sensing system consists of a substrate (for supporting sensors, fitting, and protecting human skin), a sample unit (for collecting and identifying samples), an electrode (for detecting and transmitting electrical signals), a signal unit (for conducting and amplifying signals), a data unit (for collecting, processing, and transforming data), a display unit (for displaying data analysis results), and an energy unit (for harvesting and supporting energy).^[^ ^7^ ^]^ The function of each component's material significantly impacts the performances of the components and system.^[^ ^8^ ^]^ For example, the use of materials with low sensing performances in sample units for object (gas, liquid, or mechanical signal) recognition will cause these units to fail to generate interpretable signals in time and further hinder the sensor from sensitively detecting the target object. Due to their unique application environment, the reception of signals often experiences various interferences, such as movement, sweat, and abrasion, in wearable sensing systems. Thus, signal transmission and transduction are crucial to ensure accurate, sensitive, and stable output signals for the sensing system. In this regard, electrical conductivity (EC) of a material is the foundation for guaranteeing standard signal transmission. Additionally, as wearables need to be closely attached to the human body surface, flexible and adhesive materials are required to ensure that the sensor tightly adheres to the skin surface, thereby avoiding signal distortion and interference during transmission. Finally, although hydrophobicity is not a critical factor in signal transduction, it is essential for preventing interference from sweat and aging equipment. Therefore, the development of engineered materials that possess one or more of these desired functionalities is essential for the design and manufacture of wearable sensing systems.

To meet the demands for constructing these functional materials for wearable sensors, researchers have started to seek inspiration from nature. After billions of years of interaction, sensing, and adaptation to the environment, many natural organisms have evolved complex, flexible, ingenious, and robust sensory systems. For instance, the mechanosensory lateral line system of fish can sense changes in the velocity and flow pressure of surrounding moving water, facilitating navigation and sensing of objects in the water,^[^ ^9^ ^]^ and the pinnate leaves of mimosa demonstrate tactile perception ability to sense slight pressure variations.^[^ ^10^ ^]^ Upon interaction with the real, dynamic world, biological systems exhibit advantages that electronic systems do not demonstrate; thus, the concept of bionics emerged, aiming to gain inspiration from organic organisms to solve inorganic engineering problems and promote the advancement of science and society.^[^ ^11^ ^]^ Presently, the development of functional wearable sensing materials has benefited from the biological survival wisdom and grafting of biological working principles. On the one hand, biomimetic properties endow wearable sensing‐engineered materials with more functional features. For example, the multidimensional photonic crystal structure of the butterfly provides the idea of static structural color materials^[^ ^12^ ^]^ and the chameleons inspire the development of dynamic color materials via their adaptable adjustment of the microcrystal structure arrangement in the skin iridophore layers;^[^ ^13^ ^]^ the introduction of structure forming colors principle provides a novel detection method for observing the changes in the material microstructure caused by tiny strains at the skin interface, and optical devices can be employed to notice the color changes. The hierarchical interlocked structure and tunable elastic modulus of the skin have inspired the development of wearable materials with excellent mechanical flexibilities, sensitivities, and robustness.^[^ ^14^ ^]^ On the other hand, natural organisms have afforded unique structural surface models with high detection sensitivities and multifunctional diversities. For instance, the layered porous microstructures of lotus leaves provide a superhydrophobic bionic interface model,^[^ ^15^ ^]^ the microstructures of gecko feet,^[^ ^16^ ^]^ tree frog toe pads,^[^ ^17^ ^]^ and octopus suckers^[^ ^18^ ^]^ can offer adhesive abilities to wet/dry interfaces, the mechanical hair and crack structures of spiders^[^ ^19^ ^]^ and scorpions^[^ ^20^ ^]^ are sensitive to vibration and external stimulation, and the “brick and mortar” hierarchical structure of nacre exhibits high toughness and mechanical strength.^[^ ^21^ ^]^

Application of biomimetic materials with ideal functions can further enhance the performances of wearable devices, providing strong support to these devices for achieving higher‐level perceptual abilities. Consequently, wearable artificial sensory systems (WASSs) with perceptual abilities similar to those of humans have emerged. As a biomimetic wearable sensing system with high‐level perceptual abilities, WASSs are formed by integrating high‐performance sensors and information sources. Its purpose is to simulate the human sensory system and yield more comprehensive, accurate, and real‐time perceptual data for complex tasks such as environmental perception, physiological monitoring, and motion control.^[^ ^22^ ^]^ The integration of sensors and systems has played a crucial role in the advancement of WASSs. First, in terms of integration methods, the initial independent chip‐based integration was replaced by system‐level integration because of the excessive number of components.^[^ ^7a^ ^]^ The emergence of flexible substrates has enabled sensors to adapt to different body curves and movements, providing higher‐level perception abilities including touch sensing. Second, integrated sensors have expanded from physiological to environmental, motion, and other sensors, enriching sensory scenarios.^[^ ^2c^ ^]^ Finally, in the early stages of wearable biomimetic sensory systems, sensor integration mainly focused on research and applications based on a single sensory modality. To achieve more comprehensive and realistic sensory experiences, researchers have begun to explore the integration of multiple sensors. This integration can be realized via sensor arrays, sensor networks, or modular sensor integration, all of which demonstrate advantages such as collecting multiple sensory information for comprehensive processing, enabling data sharing and collaborative work among sensors via wireless communication, and allowing combination and replacement according to user needs, thus enhancing perceptual abilities.^[^ ^7^, ^22^ ^]^ Currently, with the enrichment of substrate and sample unit material functions, system circuits, and data processing algorithms, WASSs have also developed biomimetic senses that surpass traditional human senses in some aspects, for instance, biomimetic smell that can identify dangerous and toxic gases^[^ ^23^ ^]^ and biomimetic vision that transcends the visual field of human eyes,^[^ ^24^ ^]^ which is expected to become an evolving human sense. Therefore, WASSs have become the most attractive and representative class of biomimetic wearable sensing systems.

The introduction of the bionic concept has led to significant progress in some key modules (the biomimetic materials developed to date are primarily applied to substrates,^[^ ^25^ ^]^ sample units,^[^ ^26^ ^]^ and electrodes^[^ ^27^ ^]^) and sensing methods of wearable sensing systems; however, the performances of some units of biomimetic wearable sensing systems are still limited. For example, the performance of the signal unit is limited by the slow transmission signals of complex circuits, and the performance of the data unit is restricted by the mismatch between large amounts of data and the computational power of traditional data processing technology. These limitations need to be overcome for the subsequent development of biomimetic wearable sensing systems.

**Figure 1:**
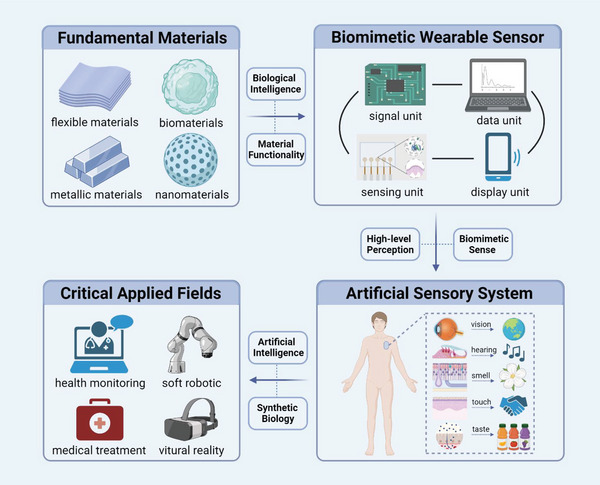
Schematic diagram of the introduction approach of biomimetic wearable sensors. Based on the four commonly used materials in wearable sensing systems, the success of biomimicry lies in enhancing the functionality of these materials to create wearable sensing systems that meet the demands of sensing. With the ability to imitate human sensory organs, artificial sensory systems offer heightened perception capabilities and can fulfill various sensing requirements. Through the integration of cutting‐edge technologies such as artificial intelligence and synthetic biology, biomimetic wearable sensors hold the potential to play a pivotal role in advanced fields. Created with BioRender.com.

In this review, five basic functional requirements (namely, sensing performance, EC, flexibility and stretchability, adhesion, and hydrophobicity) that the biomimetic materials need to satisfy for their application in wearable sensing systems are summarized. The structure of a material affects the overall performance of the material. Thus, this review outlines the enhancement effects of the introduction of biomimetic structures on the functions and functional expansion of biomimetic materials, resulting in biomimetic materials that can meet the abovementioned requirements. This review introduces WASSs from the perspective of the biomimetic senses it has developed. In addition to mimicking the five single senses, WASSs can integrate multiple biomimetic senses. To address the existing limitations of biomimetic wearable sensing systems, this review proposes a path forward that integrates artificial intelligence and synthetic biology; this path includes the use of machine learning algorithms to process a large amount of multimodal human physiological data in a short time and grafting of cell life system signal pathways to improve the efficiency of human biological signal transduction. This path can improve the sensing systems; moreover, the use of biomimetic sensing systems can help researchers synthesize high‐performance robot arms that facilitate human–machine interactions. Finally, this review presents a list of ideas and challenges in the methods of developing engineering materials and their application for the construction of target systems. The overall idea of this article is shown in **Figure** **1**.

## 2 Basic Functional Requirements that Biomimetic Materials Should Meet

### 2.1 Sensing Performance of the Sample Unit

Regarding the functional requirements of wearable sensing system materials, sensing performance is of paramount importance. When the target object reaches the sample unit, the unit material must recognize the object and convert it into interpretable signals, which is collectively referred to as sensing performance.^[^ ^28^ ^]^ Material sensing performance directly determines the outcome of molecular recognition and serves as the foundation for signal transductions. Depending on the characteristics of different detection targets, the sensing performance of the material results in distinct features, which are introduced in the following sections.

Detection of mechanical signals, including strain, stress, and mechanical vibration, in wearable forms is of significant importance for monitoring human physiological signals (such as pulses) and recognizing movements and gestures. To sense these signals, the electrical properties of the biomimetic materials of the sample units should change with respect to mechanical motion. Among the three commonly used materials for detecting mechanical signals, piezoresistive materials exhibit changes in their resistances in response to pressure or strain, piezoelectric materials generate charge separation and potential differences when subjected to pressure or stress, and frictional materials produce charge separation during friction. Representative materials for each category are graphene (resistive), piezoelectric zirconate titanate (piezoelectric), and polyvinylidene fluoride (frictional).^[^ ^29^ ^]^ Via biomimetic approaches, variations in the contact resistance between resistive materials and electrodes can be amplified,^[^ ^30^ ^]^ electromechanical polarizations of piezoelectric materials can be enhanced,^[^ ^31^ ^]^ and pulse signals of frictional materials can be modulated.^[^ ^32^ ^]^

Chemical substances, for example, alcohols, neurotransmitters, and odor molecules, in gas and liquid states play crucial roles in detecting environmental and physiological changes within the human body. To sense these chemical substances, utilization of sensing materials that can transform chemical signals into electrical signals is necessary. Commonly employed sensing materials include metal oxides, polymeric materials, and biomolecules.^[^ ^33^ ^]^ These materials can engage in specific interactions, such as electrochemical reactions, adsorption, and specific binding between antibodies and target molecules, with target chemical substances. Subsequently, they can convert the chemical signals into measurable electrical signals. The development of biomimetics has provided valuable insights into the establishment of sensing materials. By taking inspiration from the structures and functionalities of biological systems, sensing materials, such as molecularly imprinted polymers and biomimetic membranes, that resemble biological materials can be designed and synthesized.^[^ ^34^ ^]^ These materials mimic the specific recognition and transmission functions of biomolecules, thereby enabling higher selectivity and sensitivity. Furthermore, biomimetics can offer novel interfaces and interface control strategies to enhance the interactions between sensing materials and target chemical substances, thereby improving sensor performance.^[^ ^35^ ^]^

Additionally, optical sensing can be used to monitor physiological parameters including blood oxygen saturation, heart rate, and blood pressure. Optical signal sensing primarily involves photoelectric conversion and photothermal effects utilizing organic molecules, inorganic nanoparticles (NPs), and polymers. Using biomimetic materials, the light signal collection ability can be enhanced, and optical transparency can be improved.^[^ ^36^ ^]^ Furthermore, temperature sensing plays an important role in wearable sensing devices. Sensing materials for temperature signals are typically based on principles such as thermal conduction, pyroelectricity, and infrared radiation; moreover, materials typically used for this purpose include thermosensitive, thermoelectric, and infrared‐absorbing materials.^[^ ^37^ ^]^ Biomimetic materials not only mimic the thermal responses of plants and thermoregulation mechanisms of animals to detect and transform temperature changes but also simulate the structures and working principles of insect thermoreceptors to improve the sensitivities to subtle temperature variations and detection accuracies of sensing materials.^[^ ^38^ ^]^

### 2.2 ECs for the Sample Unit and Electrode

The input end of the wearable sensing system is an optical, liquid, gas, or mechanical signal. Most of these finally arrive at the signal transduction unit, such as the analog interface, in the form of an electrical signal.^[^ ^39^ ^]^ After mode transduction, the result is calculated and output to the data‐processing module. Sensitively converting external signals into electrical signals and transferring them to subsequent units is not only a test for the conversion path design but also a requirement for the ECs of the sample units and electrode materials.

**Figure 2:**
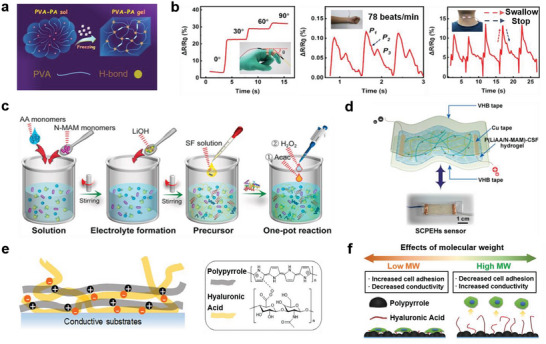
Schematic diagram illustrating the conceptual approach to fabricating biomimetic conductive materials and their characteristic features. a) Scheme for forming PVA‐PA hydrogel through a single freeze‐thaw cycle. b) Real‐time monitoring of human motion and health using PVA‐PA‐15 gel. Reproduced with permission.^[^ ^27a^ ^]^ Copyright 2019, American Chemical Society. c) The fabrication process of SCPEHs. d) Composition of sensing elements in SCPEHs sensors. Reproduced with permission.^[^ ^27b^ ^]^ Copyright 2022, Elsevier. e) Schematic diagram of PPy/HA structure. f) Schematic diagram of the effect of different molecular weights of HA on conductivity. Reproduced with permission.^[^ ^46^ ^]^ Copyright 2018, Elsevier.

Application of flexible conductive hydrogels provides a convenient way for sample units to transduce the mechanical signals from the human skin into electrical signals,^[^ ^40^ ^]^ and the use of biomimetic techniques can improve the ease of signal acquisition. In traditional methods, high filler content in an elastic body reduces the overall stretchability of the composite material.^[^ ^41^ ^]^ Therefore, researchers have transformed natural hydrogels into conductive hydrogels,^[^ ^42^ ^]^ achieving the integration of material conductivity and stretchability, facilitating the wide application of these hydrogels in flexible conductive materials of sample units.^[^ ^43^ ^]^ Nevertheless, the process involved is usually tedious, and the addition of a large amount of active electronic substances may lead to uneven distribution, further reducing the transparency of the hydrogel material. Inspired by biology, Zhang et al.^[^ ^27a^ ^]^ added phytic acid (PA), which is a common natural plant product, to poly(vinyl alcohol) (PVA) hydrogels. Via a one‐step method, they successfully developed a conductive hydrogel with outstanding properties (a large stretchability of approximately 1100% strain and high optical transparency of approximately 95%) (**Figure** **2**a), whose EC reached 0.075 S/m, enabling smooth current flow across light‐emitting diode lights. The wearable strain sensor based on the PVA‐PA hydrogel demonstrated excellent performance in real‐time human health monitoring (Figure 2b). This not only considerably simplifies the preparation of conductive hydrogels, but also offers an efficient and economical method for conductive hydrogel synthesis. Based on a similar idea, Zhou et al.^[^ ^27b^ ^]^ crosslinked natural silk fibroins and polymeric acrylic acid to construct double‐network matrices of ionic conducting silk‐crosslinked polyelectrolyte hydrogels (SCPEHs) with anti‐freezing properties (−80 °C), high mechanical strengths (0.4 MPa), and high stretchabilities (1450%) by simple and efficient one‐pot in situ synthesis (Figure 2c). Additionally, SCPEH‐based skin sensors (Figure 2d) with excellent ECs (2.58 S m^−1^) can be used to sense weak signals (such as vocal cord vibration, breathing, and heartbeat) from the human body, contributing to the advancement of subtle strain‐sensing areas that are currently underdeveloped in the field of wearable sensing.

For traditional wearable electrodes, the small sizes required for portability affect EC, whereas a biomimetic electrode constructed based on chemical modification can solve this problem to some extent. Previously, researchers have discovered that carbon nanotubes (CNTs), graphene, conductive polymers, and conductive nanowires (NWs) can be used to modify electrodes as they can form macroscopic conductive networks and enhance ECs of these electrodes; these materials are also called electrode modification materials.^[^ ^44^ ^]^ When EC is sufficient, such electrode modification materials can be directly used as electrode materials.^[^ ^45^ ^]^ However, natural sources of additives also improve the conductivities of electrodes and exhibit the advantage of appropriate biocompatibilities over the aforementioned materials. Inspired by biology, Kim et al.^[^ ^46^ ^]^ synthesized biomimetic conductive polypyrrole (PPy) films doped with hyaluronic acid (HA) of different molecular weights (Figure 2e). At frequencies lower than 100 Hz, the indium tin oxide electrodes modified with PPy/HA thin films exhibited better EC than that of the unmodified bare electrode, and the impedance magnitudes significantly decreased with an increase in the molecular weights of HA (Figure 2f). Simultaneously, the charge storage effect was improved, facilitating charge injection at the electrode/dielectric interface, allowing efficient electrical signal intermediation. Additionally, the addition of catechol, a key functional protein in mussels, enhance the ECs of pyrene‐based conductive polymers.^[^ ^47^ ^]^ In conclusion, for the sample unit/electrode material, the introduction of some active natural substances (including acids, phenols, and proteins) into the base material can effectively adjust the internal sequence of the base material and promote electron transfer.

### 2.3 Flexibilities and Stretchabilities for the Sample Unit and Substrate

As a significant sensing technology, wearable strain sensing exhibits broad application prospects in the human health monitoring and human–machine interaction fields, such as human pulse monitoring^[^ ^48^ ^]^ and speech and gesture recognition,^[^ ^49^ ^]^ and tracking sport activities, identifying postures, and monitoring physiological signals. In this regard, the flexibilities and stretchabilities of the substrate and sample unit in the sensing system are crucial for not only adapting to strain, but also enhancing the reliability and durability of the sensor. Moreover, the overall flexibility and stretchability of the sensor can improve the comfort of the wearer, thereby facilitating integration of the sensor into daily life.

**Figure 3:**
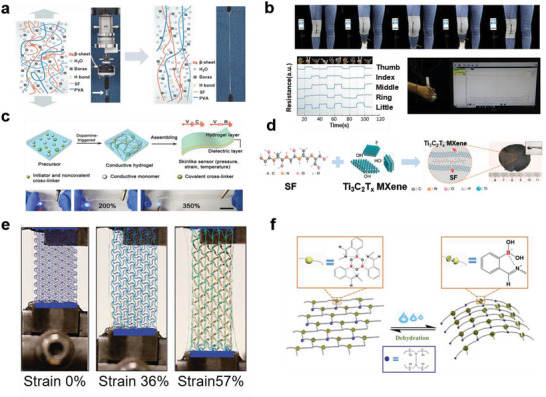
Schematic diagram illustrating the conceptual approach to fabricating biomimetic flexible and stretchable materials and their characteristic features. a) Comparison image of PVA/silk fibroin/borax (PSB) hydrogel before and after stretching. b) Strain detection application of PSB hydrogel‐based wearable sensor. Reproduced with permission.^[^ ^25a^ ^]^ Copyright 2019, American Chemical Society. c) Synthesis process (up) and flexible demonstration (down) of dopamine‐triggered gelation hydrogel. Reproduced with permission.^[^ ^52^ ^]^ Copyright 2021, American Chemical Society. d) Crosslinking process of silk fibroin and MXene molecules. Reproduced with permission.^[^ ^53^ ^]^ Copyright 2020, Elsevier. e) Morphological changes of the units in the biomimetic thin film flexible material during stretching process. Reproduced under terms of the CC‐BY license.^[^ ^55^ ^]^ Copyright 2015, Kyung‐In Jang, et al., published by Springer Nature. f) Water‐triggered self‐healing mechanism and tunable performance process of epoxy‐boron hybrid network structure. Reproduced with permission.^[^ ^56^ ^]^ Copyright 2019, American Chemical Society.

Flexible hydrogels are extensively used as substrates for wearable sensors due to their excellent biocompatibilities.^[^ ^7a^ ^]^ Biomimetic methods can further enhance the flexibilities and stretchabilities of these hydrogels while maintaining their biocompatibilities. Owing to their 3D crosslinked network structures and some natural components, for example, proteins, carbohydrates, and deoxyribonucleic acid,^[^ ^50^ ^]^ natural hydrogels demonstrate outstanding flexibilities and stretchabilities, similar to those of natural biological soft tissues. Furthermore, they exhibit better biocompatibilities than those of other common substrates such as synthetic polymers and inorganic materials;^[^ ^7a^ ^]^ therefore, they are widely used as substrates for wearable sensors. In recent years, biomimetic methods have demonstrated excellent results in terms of improving the flexibilities and stretchabilities of these hydrogels. Inspired by nature, Yang et al.^[^ ^25a^ ^]^ improved the stabilities of crosslinked networks by adding silk fibroin (a protein derived from silkworm silk fibers). Simultaneously, the polymers of PVA and borax can improve the tensile properties of the composite material, and the stretchability of the silk fibroin‐containing hydrogel reached 5000% (**Figure** **3**a). Additionally, the unique β‐sheet structure of silk fibroin helps to enhance the water retentions of PVA hydrogels, enabling the construction of an in vitro wearable sensor platform based on this hydrogel to monitor human movement and gesture for a long time with high precision (Figure 3b). A similar biomolecular additive is agar.^[^ ^51^ ^]^ Zhang et al.^[^ ^25b^ ^]^ developed a hydrogel that could demonstrate various shapes by injection; this material was based on supramolecular sodium alginate nanofibrillar. This hydrogel contained multiple hydrogen bonds and exhibited a high stretchability (3120%) and superior elasticity (100%) under a high strain (1000%). Inspired by mussels, Wang et al.^[^ ^52^ ^]^ obtained hydrogels with excellent flexibilities using dopamine as a polymerization initiator and a dynamic mediator to elaborate the hydrogel network; these hydrogels can be used as self‐adhesive substrates (Figure 3c).

For the sample unit, biomimetic approaches can change the material flexibility and modulus by adjusting the network. Wang et al.^[^ ^53^ ^]^ used natural silk fibroin as a bridging agent to promote the self‐assembly of 2D MXene nanosheets into a biomimetic crosslinked network (Figure 3d). The acquired composite film exhibited high biocompatibility, adequate flexibility, and a low elastic modulus (1.22 MPa). Additionally, the wearable pressure sensor based on this composite detected minuscule pressure changes in the human body, such as from wrist pulses, because of the high sensitivity (25.5 kPa^−1^) and low detection limit (9.8 Pa) of this composite film. Sun et al.^[^ ^54^ ^]^ reported an interesting study; they directly freeze‐dried fresh watermelon peel and then immersed it in a quantum dot dispersion to construct a MXene quantum dot/watermelon peel aerogel with a low elasticity modulus (0.03 MPa), low limit of detection (0.4 Pa), and a high pressure‐sensitive response of 323 kPa^−1^, which can be employed to monitor tiny motions, including that of pulse, and send the signal back to the Bluetooth module of the mobile phone. Jang et al.^[^ ^55^ ^]^ produced a strain‐responsive biomimetic low‐thickness film consisting of tightly connected horseshoe‐like units in a relaxed state. Under stress, its shape changes via three phases, namely, from toe to heel and linear, until plastic yielding. This multi‐shape‐changing process significantly improved the tensile limit of the material (Figure 3e). It also precisely matches the non‐linear properties of biological tissues. Inspired by the mechanical adaptabilities of sea cucumbers, Yuan et al.^[^ ^56^ ^]^ developed a dynamic, responsive material with tunable modulus, which can change from stiff to flexible by a factor of 150 under a water stimulus (Figure 3f). Dynamic flexible materials can replace traditional elastomers in the synthesis of wearable strain sensors that monitor muscle movements in the throat during the wearer's speech and demonstrate the abilities to distinguish between different audio signals from the wearer. Note that for the sample unit, although the flexibility of the material is important for sensitive strain detection, randomly employing materials with low moduli may decrease the load‐bearing capacity of this unit.

### 2.4 Adhesion for Substrate

To realize continuous signal monitoring in a wearable form, wearable sensing systems require certain adhesion of the contact interfaces related to the substrate (between the electrode and substrate^[^ ^57^ ^]^ and between the substrate and human skin^[^ ^17^, ^18^ ^]^). Some commonly used substrate materials, such as hydrogels, do not exhibit favorable self‐adhesion;^[^ ^58^ ^]^ therefore, enhancing the adhesion of substrate materials at the contact interface is necessary. The traditional method involves the addition of adhesives (including adipic‐based adhesives),^[^ ^59^ ^]^ bandages, or scotch tapes.^[^ ^60^ ^]^ However, when applied at the skin contact interface, the sensor often causes skin discomfort because of low air permeability and biocompatibility.^[^ ^61^ ^]^

**Figure 4:**
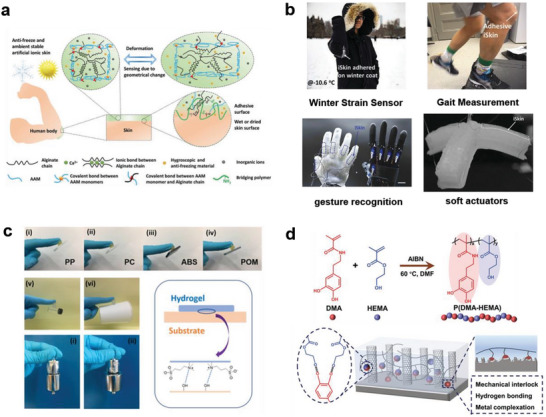
Schematic diagrams of biomimetic adhesion materials mechanisms. a) Schematic diagram of ionically crosslinked hydrogel adhering to skin substrate. b) Application of iSkin. Reproduced with permission.^[^ ^60^ ^]^ Copyright 2021, John Wiley and Sons. c) Schematic diagram of the adhesive performance and adhesion mechanism of zwitterionic nanocomposite hydrogels. Reproduced with permission.^[^ ^63^ ^]^ Copyright 2019, American Chemical Society. d) Synthesis of Poly(Dopamine Methacrylate‐Co‐Hydroxyethyl Methacrylate) material and schematic diagram of the strong adhesion mechanism with vertically aligned CNTs on the substrate. Reproduced with permission.^[^ ^64^ ^]^ Copyright 2023, John Wiley and Sons.

In this regard, biomimetic materials have been confirmed to achieve adhesive effects by mimicking the bonds generated by biomolecules.^[^ ^60^, ^61^, ^62^ ^]^ Moreover, they have attracted attention due to their excellent biocompatibilities, gentle action modes, and low skin irritation. Ying et al.^[^ ^60^ ^]^ introduced chitosan with a high concentration of primary amine groups into a hydrogel as a bridging polymer, which formed an amide bond with the to‐be‐adhered substrate (e.g., skin and carboxylated elastomer) in the presence of common coupling reagents, demonstrating improved adhesion of the hydrogel (**Figure** **4**a). By adding biocompatible cryoprotectants to mimic the wood frog, adhesion can be maintained even at temperatures below zero. The successful application of such biomimetic material in the human body and winter‐coat strain and motion/deformation sensings under extremely cold conditions for soft robots is also interesting (Figure 4b). In the biomimetic skin developed by Yang et al., the non‐covalent interaction of zwitterionic groups at the contact interface enhanced the adhesion of the hydrogel. These functional groups, namely, sulfonic acid groups and ammonium cations, provide strong bonding with diverse substances; therefore, this biomimetic skin can stably adhere to skins, plastics, glass, and steels (Figure 4c). Wearable flexible strain sensors constructed based on this substrate material are sensitive to human movements such as finger touch and speech recognition.^[^ ^63^ ^]^ Moreover, naturally regenerated silk fibroin has been proven to form a stable self‐adhesive substrate material with calcium ions and hydrogels, whose performance is better than that of commercial bandage tape.^[^ ^43c^ ^]^ Surface adhesion of a kind of copolymer can reach 6.38 MPa owing to the synergistic effects of hydrogen bonds and mechanical interlocking induced by mimicking the structures of snails and mussels; this copolymer can form a strong bond with the vertically aligned CNT (Figure 4d), which can be used for the adhesion of the electrode and substrate in the sensing system.^[^ ^64^ ^]^ Nevertheless, the adhesion performance based on chemical anchoring is susceptible to interference from various factors, for example, time, secretion, and external stimuli, and attention should be paid in this regard.

### 2.5 Hydrophobicity for the Sample Unit

**Figure 5:**
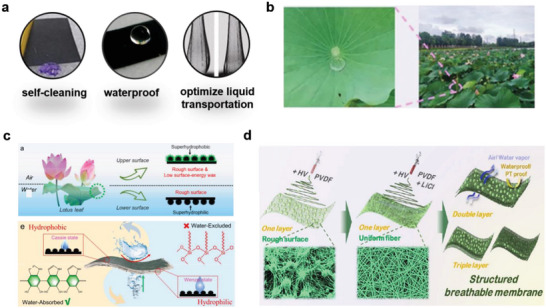
Schematic diagram of biomimetic hydrophobic materials. a) The role of hydrophobic materials in wearable systems. Self‐cleaning (left). Reproduced with permission.^[^ ^70a^ ^]^ Copyright 2018, Elsevier. Waterproof (middle). Reproduced with permission.^[^ ^15a^ ^]^ Copyright 2017, American Chemical Society. Optimize liquid transportation (right). Reproduced with permission.^[^ ^67^ ^]^ Copyright 2022, American Chemical Society. b) Real image of the lotus effect. Reproduced with permission.^[^ ^72^ ^]^ Copyright 2022, American Chemical Society. c) Schematic diagram of the superhydrophobic and superhydrophilic behavior of lotus leaves on different sides (up) and the possible mechanism of the hydrophobic/hydrophilic properties of carbon felt@AgNPs composite materials (down). Reproduced with permission.^[^ ^71^ ^]^ Copyright 2022, Elsevier. d) Design of electrospun films with controllable microbead and nanofiber structured layers. Reproduced with permission.^[^ ^72^ ^]^ Copyright 2022, American Chemical Society.

During the wearing process, the residues of the body fluids secreted by the human body can easily pollute the wearable sensor device, specifically the sampling unit, which can lead to blockage of the sample channel and the risk of degradation over time.^[^ ^26a^ ^]^ Introduction of hydrophobic materials into the sample unit can help remove the surface dirt and body fluids generated during the use of the device and prevent the unit from being corroded by metals and aging of the device, which is important for maintaining the performance and improving the durability and life of the device.^[^ ^65^ ^]^ Additionally, hydrophobic materials play critical roles in waterproofing, drag reduction,^[^ ^66^ ^]^ and improving the efficiency of liquid transport (**Figure** **5**a).^[^ ^67^ ^]^ Traditional methods for developing hydrophobic materials demonstrate drawbacks that limit their application in wearable sensing systems. For instance, the loss of material adhesion during silicone combustion^[^ ^68^ ^]^ and hydrophobic NP sprays heavily rely on expensive compounds that are harmful to the environment.^[^ ^69^ ^]^ Several hydrophobic phenomena, such as dew on distinct petals in the morning, exist in nature, which reveal that hydrophobic materials can be synthesized via biomimetic methods.

The idea of developing biomimetic hydrophobic materials originated from the lotus effect in nature (Figure 5b).^[^ ^70^ ^]^ Because of the hydrophobic characteristics of lotus leaves, the inspiration for hydrophobic coatings/materials widely arises from them. For example, inspired by the lotus leaf, the carbon felt@AgNP composite developed by Zhou et al.^[^ ^71^ ^]^ simultaneously exhibited two opposite hydration properties (hydrophobicity and hydrophilicity), and its EC was favorable (Figure 5c). A human motion monitor prepared based on this material demonstrates the unique advantage of integrated sweat removal and waterproof functions. Inspired by the bionic concept of the lotus leaf, Shi et al.,^[^ ^72^ ^]^ Chen et al.,^[^ ^73^ ^]^ and Wang et al.^[^ ^15a^ ^]^ fabricated biomimetic wearable flexible membranes with excellent mechanical properties and hydrophobicities via electrospinning/chemical vapor deposition. Shi et al.^[^ ^72^ ^]^ successfully controlled the morphologies of microbeads and nanofibers, overcoming the difficulty of using electrospinning to prepare film materials with two different morphologies (Figure 5d). Hydrophobicities of materials can also be realized by mimicking the self‐cleaning strategies of animals. Inspired by spiders, Lee et al.^[^ ^74^ ^]^ developed a hydrophobic, self‐cleaning, and stretchable conductive organogel. In nature, a spider removes contaminants by flicking its web; therefore, the authors used the wavy effect of electrostatic forces between materials to eliminate contaminants, and it took less than a minute for the organogel to return to nearly 99% of its original performance.

## 3 Material Function Enhancement through a Structural Strategy

As mentioned earlier, the biomimetic conceptual method based on the sequence‐level optimization of materials can be used to synthesize functionally engineered materials required for wearable sensing systems, whereas an ingenious material structure design is also crucial to achieve the desired function. For example, the construction of smooth conductive channels and appropriate reaction sites can enhance conductive sensitivity of the material, and control of material spacing can induce changes in its flexibility. Biomimetic structures derived from nature offer rich learning resources for the development of wearable sensing materials. For example, the crack structure is derived from the foot seams of scorpions,^[^ ^75^ ^]^ the hierarchical interlocked structure of human skin^[^ ^76^ ^]^ is sensitive to strain, the whiskers of spiders^[^ ^77^ ^]^ and cats^[^ ^78^ ^]^ can sensitively feel the external air flow and vibration, and the hierarchical structures of lotus leaves can provide superhydrophobic interfaces and self‐cleaning abilities.^[^ ^15a^ ^]^ Nacre's “brick and mortar” structures offer high toughnesses,^[^ ^21^ ^]^ while gecko pads,^[^ ^16b^ ^]^ octopus suckers,^[^ ^18a^ ^]^ and tree‐frog pads^[^ ^17^ ^]^ provide dense adhesive structures. Thus, the introduction of a biomimetic structural strategy has further optimized and enriched the functions of wearable sensing materials.

### 3.1 Multipathway Structure for Enhancing EC

**Figure 6:**
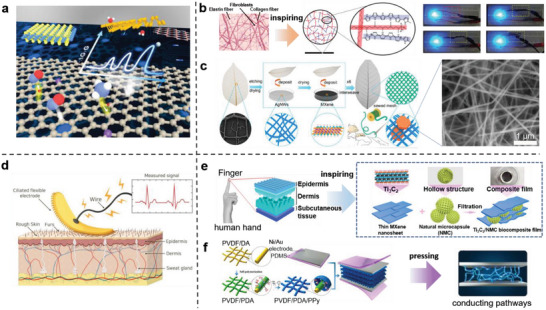
Strongly conductive materials based on biomimetic multi‐path structures. a) Schematic diagram of multi‐path structures facilitating electron transfer. Reproduced with permission.^[^ ^79^ ^]^ Copyright 2019, American Chemical Society. b) Schematic diagram of PEE‐PPy network structure, which can be used as a conductor in circuits to light up blue light emitting diode lights. Reproduced with permission.^[^ ^81^ ^]^ Copyright 2017, American Chemical Society. c) Fabrication process of AgNWs/MXene flexible transparent electrodes and scanning electron microscope (SEM) image of the conductive network. Reproduced with permission.^[^ ^83^ ^]^ Copyright 2022, American Chemical Society. d) Conceptual view of using flexible cilia electrodes to measure biological signals. Reproduced with permission.^[^ ^84^ ^]^ Copyright 2018, John Wiley and Sons. e) Inspirational schematic diagram of biomimetic Ti_3_C_2_ MXene/natural microcapsule film. Reproduced with permission.^[^ ^88^ ^]^ Copyright 2019, American Chemical Society. f) Production process and schematic diagram of pressure sensor based on 3D topological network elastic material under pressure. Reproduced with permission.^[^ ^89^ ^]^ Copyright 2020, American Chemical Society.

Under certain temperature conditions, the conductivity of a material is positively correlated with its mobility and the population of charge carriers (electrons and holes) in the internal space.^[^ ^79^ ^]^ Constructing sufficient, efficient, and stable conductive pathways can enhance the capacities and migration effects of carriers in materials,^[^ ^80^ ^]^ thereby improving their conductivities (**Figure** **6**a). Using this strategy, many biomimetic structural materials with high ECs have been developed. Inspired by the dynamic crosslinked network of animal dermis, Gao et al.^[^ ^81^ ^]^ successfully used the principle of supramolecular interactions to develop a pentaerythritol ethoxylate (PEE)–PPy hydrogel film. Due to the abundant and stable conductive pathways formed by internal hydrogen bonds and electrostatic interactions, conductivity of this film reaches 1.15 × 10^4^ S m^−1^ (Figure 6b). Leaf veins need to transport water and nutrients; thus, the surface of the leaf contains highly ordered fiber conduction networks.^[^ ^82^ ^]^ By etching this structure, Fan et al.^[^ ^83^ ^]^ successfully allowed 1D silver NWs interweave 2D MXene nanosheets to form a conductive network film with resistance as low as 0.5 Ω sq^−1^ using the capillary force generated by surface grooves (Figure 6c). Jin et al.^[^ ^84^ ^]^ proposed a flexible electrode with a biomimetic ciliary structure based on ultraconductive polydimethylsiloxane (PDMS), which can construct numerous conductive pathways between the electrode and skin cilia, thereby significantly improving the conductivity via the skin (Figure 6d). This avoids contact impedance caused by human hair on the flat electrode and motion artifacts affection when a rigid metal electrode is used. The wearable sensor based on this flexible electrode is 20% more sensitive than the latter in recognizing human heart muscle electrical signals. Additionally, researchers took inspiration from the structure of *Setaria viridis* ^[^ ^85^ ^]^ and islands^[^ ^86^ ^]^ to improve the EC of the material.

Expansion of the structural dimensions of a material can enrich the types of effective carriers,^[^ ^79^ ^]^ increase the surface‐to‐volume ratio, and provide more free electronic channels, which results in higher conductivity. The multi‐structured material can also demonstrate a larger contact area with the monitoring signal, thus improving the monitoring sensitivity of the sensor.^[^ ^87^ ^]^ Shen et al.^[^ ^88^ ^]^ mimicked the structure of human skin to develop the multidimensional structure Ti_3_C_2_ MXene/natural microcapsule film (Figure 6e); because of the synergistic effect of the interlocked structural tip contact and hierarchical structure, the resulting wearable sensor demonstrates high electron transfer efficiency and sensitivity. Compared with the planar Ti_3_C_2_ flexible sensor (2.61 kPa^−1^), the pressure sensitivity of the wearable sensor based on this material was 9.4 times higher (24.63 kPa^−1^). This sensor used to detect and distinguish sophisticated signals from finger movements and human impulses during speech recognition. Pan et al.^[^ ^89^ ^]^ synthesized a similar 3D hierarchical crosslinked structural material. Under stress, this unique structure can provide abundant contact sites for current, resulting in multidirectional conductivity enhancement (Figure 6f). Therefore, the wearable pressure sensor based on this material exhibits an extremely low detection limit (0.9 Pa), high sensitivity (139.9 kPa^−1^), fast response speed (22 ms), and cycling stability over 10 000 times. Luo et al.^[^ ^90^ ^]^ used light induction (UV irradiation) to promote the growth of a photoresponsive liquid‐crystal polymer network into jagged patterns (such as large 3D spikes) on the surfaces of fluorocarbon polymer coatings, which substantially enhanced the triboelectric effect. By coupling with PDMS substrates and Al conductive film layers, a triboelectric nanogenerator (TENG) whose open‐circuit voltage and short‐circuit current are nearly twice those of a TENG without a surface microstructure can be obtained. This TENG can be employed as a self‐powered wearable sensor to correct the shooting postures of basketball players by monitoring their movement signals during shooting.

### 3.2 Gradient Stiffness Hierarchical Structure for Obtaining Flexibility, Stretchability, and Toughness

**Figure 7:**
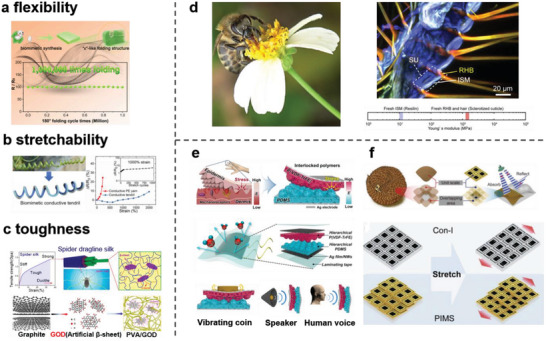
Schematic diagram of biomimetic structure optimizing mechanical properties of materials. a–c) Schematic diagrams of oriented modification of material mechanical properties by natural structures. a) Schematic diagram of using the structure of silkworm cocoons to prepare ultra‐flexible film. Reproduced with permission.^[^ ^91^ ^]^ Copyright 2021, Elsevier. b) Schematic diagram of plant‐inspired conductive tendrils with excellent stretchability. Reproduced with permission.^[^ ^95^ ^]^ Copyright 2018, American Chemical Society. c) Schematic diagram of rigid film material prepared by mimicking spider silk structure. Reproduced with permission.^[^ ^98^ ^]^ Copyright 2018, American Chemical Society. d) Honeybee foraging behavior (left) and confocal laser scanning microscope image of rigid‐flexible tongue structure (right), consisting of segmental membranes (blue) and annular bases (red/orange), characterized by flexible and rigid materials, respectively. Reproduced with permission.^[^ ^99^ ^]^ Copyright 2021, Elsevier. e) Development concept of flexible and resilient materials mimicking human skin structure (up), sensor component (middle) and their sensing applications (down). Reproduced with permission.^[^ ^76^ ^]^ Copyright 2018, American Chemical Society. f) Biomimetic mechanism of pangolin scale structure film (up) and schematic diagram comparing with conventional superstructures under stretching conditions (down). Reproduced with permission.^[^ ^102^ ^]^ Copyright 2021, John Wiley and Sons.

Regarding the mechanical properties of materials, even for detecting strain, sufficient flexibility and stretchability without a certain toughness are not satisfactory because of the difficulty in supporting more sensor parts or performing multiple cycles of load work.^[^ ^41^ ^]^ Therefore, the ideal mechanical properties are flexibility, stretchability, and toughness. In this regard, natural structural models are helpful for the directional modification of the mechanical properties of materials (**Figure** **7**a–c). The cocoon structure material can withstand 1 000 000 repeated true‐folding cycles without structural damage and can be employed to improve the flexibilities of wearable sensing materials.^[^ ^91^ ^]^ A flexible electrode with a tensile limit exceeding 70% of the uniaxial tensile strength was constructed by mimicking the lotus structure.^[^ ^92^ ^]^ The interlocked structure can endow the material with stable stretchability,^[^ ^93^ ^]^ and thin‐film patches developed using a material with a scorpion‐inspired crack structure successfully identified five different facial expressions based on the wearer's facial muscle group movements.^[^ ^94^ ^]^ The spiral structure inspired by plants tendrils^[^ ^95^ ^]^ and worms^[^ ^96^ ^]^ can be used to enhance the stretchability of the material, and then, the resulting material can be applied in wearable strain detection. To improve toughness, a wearable hydrogel‐based conductive film with a toughness of 58.9 MJ m^−3^ was fabricated by mimicking the hierarchical structure of nacre^[^ ^97^ ^]^ and breaking the bottleneck of previous double‐network crosslinked hydrogels with toughnesses of 10 MJ m^−3^. The biomimetic structure film synthesized by mimicking the nanoconfinement of hydrogen‐bonded β‐sheets in spider silk structure achieved high toughness of 30.3 MJ m^−3^.^[^ ^98^ ^]^ Is there an existing structural strategy that combines all three characteristics?

In the outstanding animal kingdom, bees can obtain nectar of varying concentrations by regulating tongue extension length, and in this process, the tongue can endure up to ten times its own weight of viscous resistance. Via further research, it was found that tongue hairs are supported by a stiff continuous ring‐like hair base and embedded in the flexible intersegmental membrane; therefore, the entire tongue is concurrently flexible and stiff. Consequently, it can simultaneously exhibit two functional properties for complete foraging (Figure 7d).^[^ ^99^ ^]^ This material structure strategy of a stiff and flexible combination is common in nature, such as in dragonflies and other insect wings^[^ ^100^ ^]^ and ladybird beetle (*Coccinella septempunctata*) setae,^[^ ^101^ ^]^ and can be employed in the development of wearable sensing materials.

Hierarchical biomimetic structures with gradient stiffnesses inspired by the stiff‐flexible combination strategy demonstrate considerable potential for application in the development engineering materials with ideal mechanical properties.^[^ ^43a,b^ ^]^ Inspired by the structure of human skin, Ha et al.^[^ ^76^ ^]^ coupled the stiff poly(vinylidenefluoride‐co‐trifluoroethylene) layer with soft PDMS layers via a hierarchical porous and interlocked microridge structure to obtain a composite. The soft microridge structure provides flexibility, and the stiff interlocked layer offers toughness. The gradient of material stiffness can guide the effective transfer of stress from the outside to the inside, whereas the interlocked and porous structure can increase the contact area and strain signal. Therefore, the wearable triboelectric sensor based on this material exhibits high sensitivity (superior to those of similar products under a pressure of 0.55 V kPa^−1^ and bending of ≈0.1 V per °) for the detection of the tiny vibrations of human vocal folds, which can be used for speech recognition (Figure 7e). Inspired by the outer hard scales and soft joint structural models of pangolins, Wang et al.^[^ ^102^ ^]^ combined a stiff electromagnetic dissipative scale with a soft elastomer (Figure 7f). Because of the flexibility of the material array joint and stiffnesses of the scales, this composite can be used in flexible sensing while maintaining the microwave interference capabilities of external scales. By mimicking the unique “brick and mortar” structure of nacre, Meng et al.^[^ ^103^ ^]^ constructed a conductive network consisting of Cu–Ag NWs and 2D reduced graphene oxide as a “brick” structure, offering EC and toughness, and infiltrated highly stretchable sodium bisulfite molecules into this network to afford flexibility and stretchability, similar to a “mortar” of nacre. The achieved strain sensor demonstrated high sensitivity (a gauge factor of up to 87362) and high break strain (up to 660%). When attached to a human–skin interface, it can respond to different movements of the face and hands.

### 3.3 Frictional Array Structure for Acquiring Long‐Term Stable Adhesion

Although bioadhesive materials that rely on molecular interactions can provide favorable adhesion to contact interfaces, most current materials exhibit limited mechanical properties of the chemical bonds formed with different surfaces, and accumulated pollutants from the skin can weaken chemical bonds or van der Waals forces. Consequently, the sensing system cannot be utilized for a long time and repeatedly;^[^ ^104^ ^]^ thus, designing structures with adhesion properties at the contact interface is a more reasonable and novel method for realizing long‐term stable adhesion. For example, to solve the adhesion problem between the substrate and electrode in a sensing system, Liu et al.^[^ ^105^ ^]^ constructed an interlocked structure between the electrode and substrate in the form of biomimetic roots, thereby increasing the adhesion between the two layers. As a proof‐of‐concept, the biomimetic electrode can be used to detect electromyography signals and monitor the deformation induced by elbow bending.

**Figure 8:**
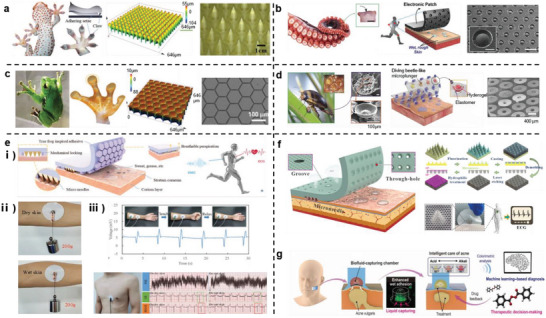
Schematic diagram of strongly adhesive material utilizing frictional array structure. a–d) Inspirations of natural structures for material adhesion. a) Microneedle array inspired by gecko claws. Reproduced with permission.^[^ ^106^ ^]^ Copyright 2022, John Wiley and Sons and Reproduced with permission.^[^ ^26b^ ^]^ Copyright 2022, American Chemical Society. b) Suction cup array inspired by octopus tentacles. Reproduced with permission.^[^ ^18b^ ^]^ Copyright 2018, John Wiley and Sons. c) Hexagonal array inspired by tree frog footpads. Reproduced with permission.^[^ ^102^ ^]^ Copyright 2022, John Wiley and Sons and Reproduced under terms of the CC‐BY license.^[^ ^17^ ^]^ Copyright 2022, Kyung‐In Jang, et al., published by John Wiley and Sons. d) Plunger array inspired by the spatula setae of diving beetles. Reproduced under terms of the CC‐BY license.^[^ ^61^ ^]^ Copyright 2021, Sangyul Baik, et al., published by American Association for the Advancement of Science, AAAS. e) Strongly adhesive patch combining gecko and tree frog biomimetic structures. i) Composition, ii) Adhesive performance, and iii) Applications for detecting electromyographic and electrocardiographic signals. Reproduced with permission.^[^ ^102^ ^]^ Copyright 2022, John Wiley and Sons. f) Multi‐biomimetic patch with wedge‐shaped channels based on conical hole structure, and its applications. Reproduced with permission.^[^ ^26b^ ^]^ Copyright 2022, American Chemical Society. g) Principle of adhesive patch inspired by diving beetles and its practical application for analyzing pH values of unstained skin. Reproduced under terms of the CC‐BY license.^[^ ^61^ ^]^ Copyright 2021, Sangyul Baik, et al., published by American Association for the Advancement of Science, AAAS.

To address the adhesion problem between the bottom substrate layer of the sensor and human skin, the main structural strategy is to engraft a biomimetic structure of a biocharacteristic adhesion pattern array on the substrate layer, which can be summarized as a structure with frictional arrays. The inspirations are primarily taken geckos (microneedle‐shaped structures),^[^ ^106^ ^]^ octopuses (suction‐cup‐shaped structures),^[^ ^18b^ ^]^ tree frogs (hexagon‐shaped structures),^[^ ^104^ ^]^ and diving beetles (plunger‐shaped structures)^[^ ^61^ ^]^ (**Figure** **8**a–d). Compared to the adhesion problem under dry conditions, in the field of flexible wearable electronics, the issue of wet surface adhesion has become more prominent due to the accumulation of sweat, mucus, and even blood on the surfaces of biological tissues.^[^ ^104^ ^]^ While maintaining the focus on the dry/wet adhesion properties of the long‐studied micro/nano biomimetic adhesive structures of gecko^[^ ^107^ ^]^ and octopus,^[^ ^108^ ^]^ several groups have recently identified the unique wet‐adhesion advantages of emerging biomimetic interfaces inspired by tree frog toe pads. For example, Chen et al. discovered that arrays of microcolumns mimicking the toe pads of tree frogs triggered liquid self‐adjustment in wet environments, promoting the generation of nanometer‐thick liquid bridges with strong boundary frictions at the microcolumn boundary.^[^ ^104^ ^]^ Therefore, the biomimetic structure exhibits strong boundary friction, which is approximately 20 times the wet friction without an external force. This avoids the problem of requiring the pre‐addition of force, which can easily damage the wearer's soft tissue, reported in previous research based on the structure of the octopus sucker. Ji et al. applied the adhesion of the microstructures of tree frog toe pads to a wet substrate and combined the resulting substrate with a gecko‐inspired microneedle array; thus, the adhesion of the interface was further enhanced owing to mechanical locking ability of the microneedle array.^[^ ^106^ ^]^ The corresponding biomimetic electrode can withstand 200 g of vertical tension at the dry/wet interface and continuously monitor electrocardiographs and electromyograms on the human body skin (Figure 8e). Based on this study, a biomimetic conical hole structure was introduced (Figure 8f). Under the action of the Laplace liquid pressure difference generated by the wedge channel, the liquid can be transferred from the skin to the air in a directional manner, thereby rendering the patch breathable. The adhesion of the biomimetic array ensures the stability of signal acquisition of the bionic health‐monitoring electrode patch, providing a novel method for the accurate monitoring of bioelectrical signals during skin perspiration.^[^ ^26b^ ^]^

Additionally, a biomimetic patch based on the structure of an octopus sucker has been successfully exploited to sustain weight (500 g) underwater;^[^ ^18b^ ^]^ however, the probable damage caused by excessive adhesion to the wearer's skin during separation should be considered. Using similar bottom sucker structures, Baik et al.^[^ ^61^ ^]^ developed flexible sweat‐collecting patches with structures that mimicked the suction plungers in the setae of male diving beetles. These specific cavity structures not only can robustly and reversibly adhere to irregular human skin under dry/wet conditions, but can also capture the water in the cuticle of human skin using the embedded biological fluid hydrogel. By combining machine learning algorithms, the water pH on the skin surface can be monitored, which is helpful for the early diagnosis and treatment of skin diseases (Figure 8g).

### 3.4 Micro/Nanoprotrusion Structure for Exhibiting Superhydrophobicity

**Figure 9:**
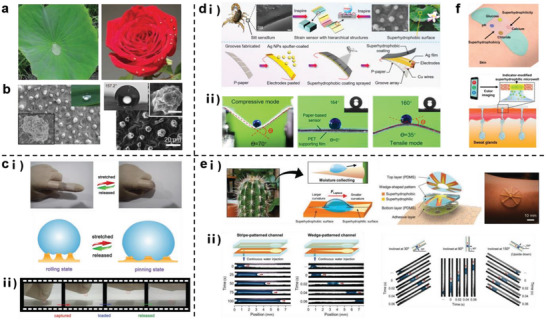
Schematic diagram of the principle and application of biomimetic superhydrophobic material based on micro‐nanoprotrusion structures. a) Prototype of superhydrophobic structures in nature (using lotus leaves and rose petals as examples). Reproduced with permission.^[^ ^110^ ^]^ Copyright 2018, John Wiley and Sons. b) Schematic diagram of micro‐protrusion structures on lotus leaves (left). Reproduced with permission.^[^ ^15a^ ^]^ Copyright 2017, American Chemical Society and the superhydrophobicity of biomimetic structural materials (right). Reproduced under terms of the CC‐BY license.^[^ ^113^ ^]^ Copyright 2022, Wenjian Li, et al., published by Elsevier. c) Wearable skin patch with switchable wettability. i) Schematic diagram of the conversion mechanism and ii) differential wettability performance. Reproduced with permission.^[^ ^18b^ ^]^ Copyright 2018, John Wiley and Sons. d) Wearable paper‐based strain sensor with biomimetic structures inspired by scorpions and lotus leaves. i) Production process and ii) superhydrophobic performance under different strain conditions. Reproduced with permission.^[^ ^114^ ^]^ Copyright 2021, American Chemical Society. e) Biomimetic superhydrophobic––superhydrophilic sweat sensor. i) Production process, composition, appearance, and ii) sweat transport capacity. Reproduced with permission.^[^ ^26a^ ^]^ Copyright 2021, John Wiley and Sons. f) Conceptual diagram of a microwell‐type sweat delivery skin bandage as a platform for sweat sampling and monitoring. Reproduced with permission.^[^ ^118^ ^]^ Copyright 2019, American Chemical Society.

Unlike hydrophobic coating materials, which rely on the surface biochemical reaction or physical action,^[^ ^109^ ^]^ hydrophobic structural materials retain the hydrophobic state of the interface via water repellence. This milder structural strategy can be considered while designing structures with densely packed micro/nano scale protrusions. Numerous superhydrophobic structural models, such as lotus leaves^[^ ^109a^ ^]^ and rose petals,^[^ ^110^ ^]^ exist in nature (**Figure** **9**a). By emulating these structures, micro/nano protrusions can be fabricated on material surfaces, which prevent complete wetting by water droplets and confer superhydrophobicity, typically characterized by a water contact angle exceeding 150° and a water sliding angle lower than 10° (Figure 9b).^[^ ^109b^ ^]^ Wang et al.^[^ ^110^ ^]^ synthesized a superhydrophobic skin‐like stretchable surface based on materials that could switch their structures between lotus leaf and rose petal structures. When integrated into a skin‐like device worn over the finger, the surface exhibited water repellency due to its lotus leaf‐like superhydrophobic structure. Nevertheless, wearers can switch the surface morphology of the structure by bending the joints, thereby increasing the water–solid contact area and generating petal‐like superhydrophilic properties (Figure 9c). Compared with the study of Shao et al.,^[^ ^111^ ^]^ which required heating to trigger a hydrophilic‐hydrophobic state change, this dynamic switching mode combined with finger movement does not need external energy and is more suitable for the human body, which is expected to be used in emerging wearable applications.

Additionally, in practical wearable sensor applications, the performance of the sensing system is reduced by contamination with sweat produced by the human body,^[^ ^112^ ^]^ whereas biomimetic superhydrophobic structures demonstrate self‐cleaning and pollution resistance abilities. Li et al.^[^ ^113^ ^]^ developed a bioinspired sweat‐resistant wearable TENG based on a material that mimicked the hierarchical micro‐nanostructures of lotus leaves, exhibiting excellent anti‐pollution and anti‐humidity properties. After introducing 0.9% saline and 70% relative humidity, the output loss of the flat TENG was higher than 40% when compared with that of the bioinspired TENG, and the bioinspired TENG demonstrated stability even under extreme pollution and humidity conditions. The bioinspired TENG maintained stable performance after the wearer completed a series of sweaty movements. With regard to lightweight and green electronic products, paper sensors have attracted attention because of their light weights and environmental characteristics; however, their applications are limited by their lower performances when they touch water and therefore cannot be widely used in wearable interfaces. Liu et al.^[^ ^114^ ^]^ integrated a crack structure inspired by scorpions and a superhydrophobic structure inspired by the lotus leaf on a paper‐based wearable strain sensor material in a staggered arrangement (Figure 9d). The sensor exhibited a gauge factor of 263.34 and stability over 12000 cycles while maintaining an excellent water resistance of 164°, which substantially improves the application prospects of paper‐based sensors in wearable interfaces. Furthermore, superhydrophobicity can be introduced into a superhydrophilic interface to improve the liquid transport efficiency via a reasonable biomimetic structure design (specifically suitable for wearable sweat‐sensing electronics).^[^ ^115^ ^]^ A typical biomimetic structural design is the creation of wedge‐shaped liquid channels (inspired by Cactus,^[^ ^26^, ^116^ ^]^ *Nepenthes Alata*,^[^ ^117^ ^]^ and Sarracenia^[^ ^65a^ ^]^) for applications in wearable body fluid sensors. Centripetal directional Laplace pressure (P) induced by the wedge channel shape (geometric structure with curvature radius ranging from small to large) (Equation 1) and the driving force generated by the difference between the water affinities of adjacent interfaces (superhydrophobic/superhydrophilic) can coordinate the directional transport of liquid and improve the sweat collection rate. This structural material design can maintain the areal hydrophobic state and efficient liquid transport and is a popular design in wearable body fluid sensing.

(1) $$P=\gamma \left(\frac{1}{R_{1}}+\frac{1}{R_{2}}\right)$$

Compared to traditional microfluidic technology, this biomimetic structure does not need to wait for sweat to fill the entire channel area (Figure 9e), which is more efficient. Moreover, the Laplace pressure induced by the physical structure delivers the liquid, which is more energy efficient. Simultaneously, it can reduce the adverse impact of bacterial and biochemical degradation caused by residual sweat on the sensor life. For example, Son et al.^[^ ^26a^ ^]^ used a cactus‐spine‐inspired wedge‐shaped liquid transport channel in the hierarchical micro/nanostructure of a skin patch and superhydrophobic and superhydrophilic materials on two sides of the channel interface. Compared with conventional microfluidic channels, this channel can realize non‐residual droplet delivery, exhibit thousands of times higher efficiency, and can even reverse‐absorb sweat when the channel is vertical to the ground. This design solves many problems, including mixing old sweat with new sweat and gathering low sweat concentrations for detection, in sensing sweat. This patch‐based sensor can quickly respond to and continuously monitor biochemical substances in the sweat of a wearer (Figure 9e). Similar biomimetic liquid channel designs for wearable sweat sensors and microwell shapes^[^ ^118^ ^]^ also require propulsion assisted by hydrophobic interactions between the superhydrophobic and hydrophilic interfaces (Figure 9f). Superhydrophobic materials based on micro/nano protrusions have demonstrated significant self‐cleaning and directional liquid transport abilities in sensors. To further optimize and enhance the performances of these wearable engineering materials, advanced fabrication techniques can be employed to improve their mechanical durabilities and expand their application scope such as in anti‐icing, oil‐water separation, and drag reduction.

### 3.5 Layered Structure for Generating Electromagnetic Interference (EMI) Shielding

With the development of wearable sensing devices, materials are required to not only be flexible and conductive, but also exhibit the ability to shield EMI to protect sensitive electronic device components from damage and wearers from radiation contamination. Currently, extensive interest is being directed toward the development of layered biomimetic structures as EMI shielding materials/coatings. This approach enhances the EMI shielding effectiveness of the material via multiple mechanisms, including multiple reflections and scattering, absorption and dissipation, lengthening of the propagation path, and matching of the electromagnetic wave wavelength with the interstitial size, while maintaining the mechanical flexibility and multiple signal stimulation conductivity of the material.

**Figure 10:**
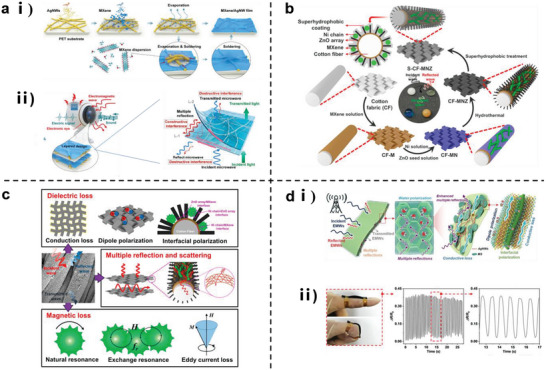
Schematic diagram of layered structure material with electromagnetic shielding interference capability. a) Schematic diagram of layered AgNWs/MXene thin film. i) Fabrication process and ii) electromagnetic shielding principle. Reproduced with permission.^[^ ^119^ ^]^ Copyright 2020, American Chemical Society. Layered MXene/Ni chain/ZnO array cotton fabrics, b) fabrication process and c) electromagnetic shielding interference mechanism. Reproduced with permission.^[^ ^120^ ^]^ Copyright 2020, American Chemical Society. d) Honeycomb structure hydrogel. i) Electromagnetic shielding mechanism and ii) application for strain detection. Reproduced with permission.^[^ ^122^ ^]^ Copyright 2022, American Chemical Society.

Generally, the electromagnetic wave pass rate strongly depends on the density of the conductive material; thus, the hierarchical structure of lotus leaf has important implications for the development of EMI shielding materials. This is because these structures can enhance EMI shielding effectiveness by increasing the times of electromagnetic reflection‐absorption inside the material, as demonstrated in a carbon felt@Ag composite inspired from the lotus leaf structure, exhibiting an excellent EMI shielding effectiveness of 65 dB at a thickness of only 270 µm.^[^ ^71^ ^]^ Chen et al.^[^ ^119^ ^]^ and Wang et al.^[^ ^120^ ^]^ applied Ti_3_C_2_T*_x_* MXene, a recently discovered micro/nanoscale wearable flexible EMI shielding material,^[^ ^121^ ^]^ as a coating on metal NW networks to construct flexible hierarchically structured materials. The materials exhibited EMI shielding performances of 49.2 and 35.1 dB (**Figure** **10**a–c). Practical effects were verified by preparing a wearable acoustic sensor film and fabric. According to Chen et al.,^[^ ^119^ ^]^ the hierarchical structures of materials can improve the EMI shielding performances without significantly sacrificing visible transmittance.

Although the idea of relying on a high material density is valid, other biomimetic structures have been reported to confer EMI shielding capabilities. For example, inspired by nature, the honeycomb‐like structure hydrogel prepared by Yang et al.^[^ ^122^ ^]^ is not only stable, but also exhibits high EMI shielding effectiveness that reaches 90 dB in the X band. This design can significantly improve the sensitivity and safety of the wearable human movement monitoring device based on it (Figure 10d). In the study reported by Wang et al.,^[^ ^123^ ^]^ the added AgNPs not only coated conductive nanofibers to form an electromagnetic shielding layer of the core‐shell structure, but also enhanced the conductivity, thus overcoming the problem that the traditional dosage of conductive filler compound is extremely high to adapt to comfortable wearing. Additionally, the superhydrophobic interface introduced into this composite enhances the electromagnetic shielding interference ability of this wearable flexible material in a wet environment, and EMI shielding effectiveness of the produced 0.06 mm thin film can reach 82.60 dB.

Biomimetic materials exhibit significant promise for electromagnetic shielding in wearable electronic devices. Natural structures, such as lotus leaves, honeycombs, and exoskeletons, have been proven effective in shielding electromagnetic waves. This has inspired the development of biomimetic materials with excellent electromagnetic shielding properties. By combining different materials and regulating the structures of the resulting materials, the application ranges and performances of these materials can be continuously expanded, providing efficient and reliable solutions for electromagnetic shielding in wearable electronic devices.

## 4 High‐Level Perception System to Achieve Artificial Sensation

Biomimetic materials with better functionalities can be designed and fabricated, offering a foundation for the construction of advanced biomimetic wearable sensors. These sensors utilize the principles of biomimicry to simulate human perception mechanisms and achieve higher‐level recognition abilities.^[^ ^22^, ^124^ ^]^ Key sensing and signal transduction units in the system are critical for achieving different high‐level biomimetic sensations. Notably, in the era of big data, traditional electronic devices struggle to sense neuronal signals, rendering the development of biomimetic neuromorphic electronic devices with synaptic functions necessary.

### 4.1 Tactile Perception

As an important sensory ability of the human body, tactile perception begins with skin receptors, including low‐threshold mechanoreceptors and free nerve endings, that receive direct stimulation from the external environment and ends after the central nervous system processes the signals conducted by internal nerve fibers. Because of the high efficiency, sensitivity, adaptability, and natural biocompatibility brought to biological skin tactile sensing systems by the evolution of species, considerable efforts have been made to explore the fabrication of wearable tactile sensing electronic devices based on the bionic perspective, and to date, significant progress has been made in material functionality, sensitivity,^[^ ^125^ ^]^ detection range, and other aspects.^[^ ^126^ ^]^

**Figure 11:**
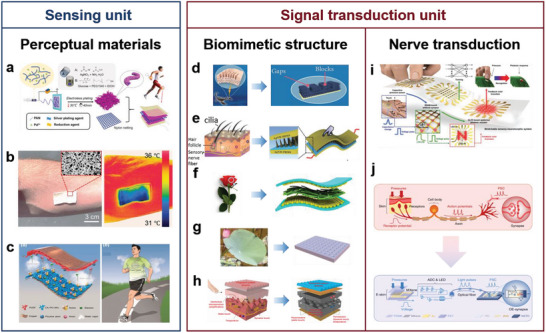
Schematic diagram of biomimetic tactile sensors. a–c) Basic materials of sensing units in biomimetic tactile sensors. a) Pressure‐sensitive materials for sensing pressure signals. Reproduced with permission.^[^ ^127a^ ^]^ Copyright 2020, Elsevier. b) Thermoelectric materials for sensing temperature signals. Reproduced with permission.^[^ ^196c^ ^]^ Copyright 2023, John Wiley and Sons. c) Electromechanical materials for sensing mechanical signals. Reproduced with permission.^[^ ^112a^ ^]^ Copyright 2017, Elsevier. d–h) Biomimetic structures for improving signal transduction sensitivity in tactile sensing. d) Spider‐inspired crack microstructure. Reproduced under terms of the CC‐BY license.^[^ ^75^ ^]^ Copyright 2021 Shengshun Duan et al., published by American Association for the Advancement of Science, AAAS. e) Human cilia‐inspired hair‐like microstructure. Reproduced with permission.^[^ ^133a^ ^]^ Copyright 2019, Royal Society of Chemistry. f) Rose petal‐inspired layered microstructure. Reproduced with permission.^[^ ^130b^ ^]^ Copyright 2021, American Chemical Society. g) Lotus leaf‐inspired porous microstructure. Reproduced with permission.^[^ ^135a^ ^]^ Copyright 2018, John Wiley and Sons. h) Human skin‐inspired interlocking microstructure. Reproduced under terms of the CC‐BY license.^[^ ^125^ ^]^ Copyright 2015, Youngoh Lee, et al., published by American Association for the Advancement of Science, AAAS. i–k) Neural conduction in biomimetic tactile signal transmission. i) Sensory neuromorphic system inspired by the golden tortoise beetle. Reproduced with permission.^[^ ^143^ ^]^ Copyright 2021, John Wiley and Sons. j) Tactile sensing artificial afferent nerve system integrated with open‐eye synapse. Reproduced under terms of the CC‐BY license.^[^ ^144a^ ^]^ Copyright 2020, Hongwei Tan, et al., published by Springer Nature.

Sensing units of biomimetic wearable tactile sensors comprise various materials, for instance, piezoresistive, thermoelectric, and electromechanical materials, which can perceive different tactile signals such as pressure, temperature, and vibration (**Figure** **11**a–c). Particularly, piezoresistive materials, including conductive polymers and CNTs, can precisely detect changes in pressure and object deformation,^[^ ^127^ ^]^ whereas thermoelectric materials, such as inorganic semiconductors and organic materials, can measure subtle temperature variations.^[^ ^128^ ^]^ Electromechanical materials, for example, piezoelectric and frictional materials, can detect the frequencies and amplitudes of mechanical vibrations.^[^ ^29^, ^129^ ^]^ Moreover, multifunctional materials, including graphene, not only can sense multiple tactile stimuli, such as pressure and temperature changes, but can also perform high‐precision detection and analysis of the shapes, textures, and surface properties of objects at the microscale.^[^ ^39a^ ^]^ Development of these different sensing units and materials offers ample opportunities for wearable devices to mimic human touch and create richer, more refined, and intelligent user experiences in several application areas, for instance, healthcare and human–machine interaction.

However, the current challenge in this field is to obtain more sensitive sensing electronics with wide linear detection ranges to satisfy the demands of heart pulse monitoring, brain environmental monitoring, soft robotics, and other fields for monitoring subtle signals (usually below 10 kPa).^[^ ^130^ ^]^ Microstructures of materials have been proven to be of significant importance in enhancing their responses to tactile signals.^[^ ^131^ ^]^ Recently, biomimetic structures such as cracked,^[^ ^75^, ^132^ ^]^ hair‐like,^[^ ^133^ ^]^ hierarchical,^[^ ^130^, ^134^ ^]^ porous^[^ ^45^, ^135^ ^]^ and interlocked^[^ ^136^ ^]^ structures have considerably improved the sensitivities of materials under different pressure conditions and extended the linear detection range by altering the contact area and electronic transfer efficiency of the active material (Figure 11d–h). Lee et al.^[^ ^137^ ^]^ fabricated a tactile sensor by combining multi‐bionic structures. Based on the geometric structure of the interlocked microdome, they set hierarchical structures inside the microdome with stiffness and conductivity gradients between different layers. The stiffness gradient can determine the stress location during the transfer of tactile stress to the sensing area. The conductivity gradient can activate the current pathways from the outer to inner layers and selectively regulate current transfer, resulting in sensitive piezoresistive changes. The sensor exhibits an extremely high‐pressure sensitivity (3.8 × 105 kPa^−1^), a wide detection linear range (100 kPa), a fast response time of 0.016 ms, and a minimum detectable pressure level of 0.025 Pa. Another biomimetic interlocked tactile sensor developed via photopolymerization shrinkage demonstrates an extensive pressure range (0.6–2 MPa) with a high sensitivity (348.28 kPa^−1^).^[^ ^138^ ^]^ Therefore, enhancing the sensitivities of materials by endowing them with biomimetic structures that can provide enriched conduction pathways is of considerable significance.

Neural morphological components based on materials capable of effectively sensing and transducing signals have substantially contributed to the signal transduction of artificial tactile systems, which can achieve higher levels of perception by imitating the human tactile perception mechanism. Intelligent skin components such as ion channels,^[^ ^139^ ^]^ mechanoreceptors,^[^ ^140^ ^]^ and sensory neurons^[^ ^141^ ^]^ have made bionic tactile functions possible; however, the integration of full touch‐sensing components is essential.^[^ ^142^ ^]^ Recently, Kim et al.^[^ ^143^ ^]^ synthesized a novel integrated wearable and stretchable sensory neuromorphic system (SSNS) that included an artificial pressure receptor, artificial synapse, and epidermal photonic driver (Figure 11i). The artificial synapse can transform mechanical signals into electrical signals and interact with neurons, enabling the construction of an artificial neural network. The successful development of SSNS affords new ideas and models for wearable smart skin (Figure 11j).^[^ ^144^ ^]^

### 4.2 Olfactory Perception

Artificial noses based on olfactory bionics have a history of more than 40 years, and the representative product is an electronic nose.^[^ ^145^ ^]^ Nevertheless, traditional electronic nose devices demonstrate the disadvantages of large sizes, difficulties in portability, and limited application scenarios. In contrast, emerging wearable biomimetic olfactory sensors can be employed for the recognition of dangerous gases,^[^ ^23^, ^146^ ^]^ food quality identification^[^ ^23^, ^147^ ^]^ and medical diagnosis^[^ ^124^, ^148^ ^]^ and are preferred by people due to the advantages of portability and diverse application scenarios.

**Figure 12:**
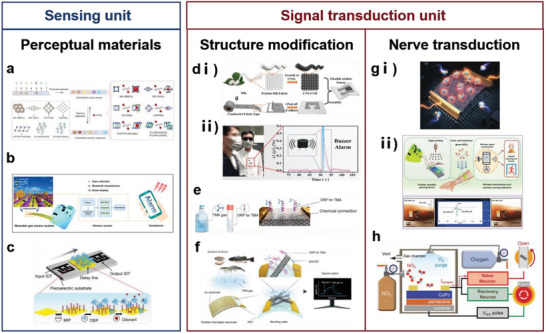
Schematic diagram of biomimetic olfactory sensors. a–c) Basic materials of sensing units in biomimetic olfactory sensors. a) Chemical adsorption materials. Reproduced with permission.^[^ ^147b^ ^]^ Copyright 2022, Elsevier. b) Physical adsorption materials. Reproduced with permission.^[^ ^149b^ ^]^ Copyright 2022, American Chemical Society. c) Organic biological materials. Reproduced under terms of the CC‐BY license.^[^ ^124^ ^]^ Copyright 2022, Chuanting Qin et al., published by John Wiley and Sons. d–f) Material structure modifications for improving odor sensing ability of olfactory sensors. d) Fluffy‐like CNT‐modified wearable olfactory biomimetic sensor, i) fabrication process, and ii) application for blind people to perceive surrounding airflow. Reproduced with permission.^[^ ^152^ ^]^ Copyright 2020, John Wiley and Sons. e) Olfactory biomimetic sensor connecting ORPs and single‐walled CNTs by chemical means. Reproduced with permission.^[^ ^23b^ ^]^ Copyright 2022, Elsevier. f) Effective connection of ORP increased by 3D bending structure and used for detecting trimethylamine content in food. Reproduced with permission.^[^ ^23c^ ^]^ Copyright 2022, American Chemical Society. g) Artificial neuron‐like gas sensor based on CuS Quantum Dots/Bi_2_S_3_, i) gas adsorption schematic, and ii) application process for visual detection of NO_2_. Reproduced under terms of the CC‐BY license.^[^ ^156^ ^]^ Copyright 2021, Xinwei Chen et al., published by Springer. h) Schematic diagram of NO_2_ sensing system based on sensory synapses and neural circuitry. Reproduced with permission.^[^ ^155a^ ^]^ Copyright 2022, John Wiley and Sons.

Sensing unit of a wearable biomimetic olfactory sensor typically comprises two parts: a sensing chip and recognition material. Common recognition materials for gas sensing include inorganic adsorption and organic biological materials (**Figure** **12**a–c). Inorganic materials typically consist of chemical and physical adsorption materials that generate distinguishable signals by adsorbing gases on the their surfaces. Although chemical adsorption materials exhibit favorable selectivities and stabilities, they require activation energy and demonstrate slow kinetics. Examples of these materials are metal or semiconductor NPs and metal‐organic frameworks.^[^ ^40^, ^147^ ^]^ Although physical adsorption materials exhibit fast kinetics, they suffer from lower selectivities and stabilities. Examples of these materials include metal‐oxide semiconductors and semiconductor quantum dots.^[^ ^149^ ^]^ Organic biological materials typically involve odorant receptor proteins,^[^ ^150^ ^]^ which sense the interaction between target gas molecules and receptor proteins, triggering conformational changes in the receptor proteins, thereby changing the electrical or optical properties of the sensing chip for odor recognition.

For the sensing unit, regardless of whether inorganic or organic materials are used, adjustment of the material structure to expose maximum effective binding sites for gas molecules is of considerable significance for improving the recognition capability of this unit.^[^ ^151^ ^]^ Wang et al.^[^ ^152^ ^]^ developed an all‐textile airflow sensor modified with spider fluff‐like CNTs. With enhanced volatile organic compound (VOC) contact area, this sensor exhibits an ultra‐low detection limit (≈0.05 m s^−1^) and multiangle airflow differential response (0–90°). Particularly, when flexible gas sensors are integrated into human clothing, they can monitor changes in airflow velocity in the environment and inform blind people of potential hazards via Morse code of letters (Figure 12d). Huang et al.^[^ ^153^ ^]^ have discovered that the Lepidoptera scale‐like gas sensor demonstrates a lower detection threshold of 0.0023 m s ^−1^ than that of an insect fluff‐like structure, which is lower than that reported in the literature, and the Lepidoptera scale‐like gas sensor can be successfully applied in communication for quadriplegic aphasia. Qin et al. chemically conjugated olfactory receptor‐derived peptides (ORPs) to single‐walled CNTs modified with thioesters (Figure 12e)^[^ ^23b^ ^]^ via a 3D bending structure design of the electrode (Figure 12f),^[^ ^23c^ ^]^ increasing the effective connection amounts of ORPs by 4.7 times. The biomimetic gas sensor improved the limit of detection for gaseous trimethylamine from the reported lowest limit of 10 parts per quadrillion (ppq) to 0.1 ppq and can be used to monitor whether food (such as fish and beef) is spoiled. Compared with inorganic adsorption materials, these materials demonstrate better selectivities;^[^ ^154^ ^]^ however, the introduction of receptor proteins simultaneously poses potential threats to sensor performance because of environmental factors (temperature, pH, and metal ions).

Biomimetic artificial neural networks are an emerging way of olfactory sensing information transduction, capable of achieving rapid unbiased recognition and parallel processing of unstructured gas data.^[^ ^155^ ^]^ Yang et al. reported a group of wearable biomimetic olfactory gas sensors inspired by the neuron conduction mechanism for the first time.^[^ ^149^, ^156^ ^]^ They employed Au quantum dots to create large amounts of sulfur vacancies evenly distributed on the surfaces of Bi_2_S_3_ nanosheets, which were used as artificial sensory neurons to capture specific gas molecules, and the Bi_2_S_3_ nanosheets acted as efficient current‐transfer channels to realize rapid charge transfer.^[^ ^149b^ ^]^ The lowest response value of this wearable device to NO_2_ was extremely low (5.6–5 ppm); nevertheless, they used CuS quantum dots to replace Au quantum dots, further reduced the theoretical detection limit of NO_2_ (0.078 ppm), and realized the visual detection of NO_2_ (Figure 12g).^[^ ^156^ ^]^ The artificial synapses developed by Qian et al.^[^ ^155a^ ^]^ and Li et al.^[^ ^155b^ ^]^ can also exhibit bionic behaviors (including excitation and inhibition) for specific gases (such as NH_3_ and NO_2_) in the sensing system and convert the responses into interpretable electrical signals (Figure 12h).

### 4.3 Gustatory Perception

Taste detection plays an important role in public safety, food screening, and human health. Traditional sensory analysis methods are not widely used due to ethical constraints and individual differences in taste. Therefore, fabrication of in vitro taste bionic systems is necessary. Presently, wearable biomimetic taste sensors can easily and accurately simulate natural taste, beyond earlier electronic tongues, owing to their advantages of high sensitivities, specificities, and miniaturization.^[^ ^157^ ^]^ When applied on wearable devices, they are expected to compensate for the loss of relevant sensory functions in coronavirus disease 2019 patients. To some extent, it can reduce the risk of ingesting toxic substances due to lack of taste during illness.

**Figure 13:**
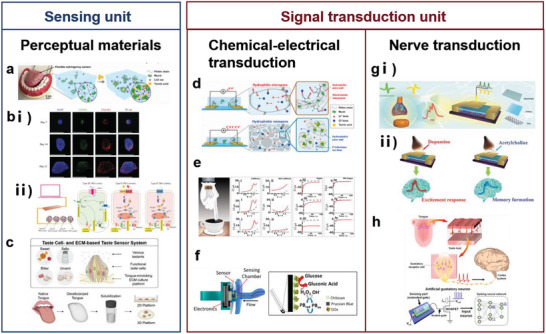
Schematic diagram of biomimetic gustatory sensors. a) The material composition of the sensing element of the flexible biomimetic gustatory sensor includes a loaded mucin protein. Reproduced under terms of the CC‐BY license.^[^ ^158a^ ^]^ Copyright 2020, Jeonghee Yeom et al., published by American Association for the Advancement of Science, AAAS. b) An in vitro taste organoid system with self‐renewal capability. i) Immunostaining images of key taste receptor proteins (otopetrin‐1 and taste receptor type 1 member 2) on the taste organoid cultured for 7, 14, or 21 days and ii) schematic illustration of the taste organoid's ability to detect sour, sweet, bitter, and salty stimuli. Reproduced under terms of the CC‐BY license.^[^ ^158b^ ^]^ Copyright 2023, Jianguo Wu et al., published by John Wiley and Sons. c) Schematic illustration of the taste sensing system based on taste cells and the extracellular matrix of the tongue. Reproduced with permission.^[^ ^160^ ^]^ Copyright 2018, Elsevier. d) In the flexible biomimetic tannic acid sensor, the working principle is based on the recognition of tannic acid by mucin to generate a detectable electrical signal. Reproduced under terms of the CC‐BY license.^[^ ^158a^ ^]^ Copyright 2020, Jeonghee Yeom et al., published by American Association for the Advancement of Science, AAAS. e) A robot electronic skin based on the chemical‐electrical conversion principle can detect caffeine and sugar in beverages. Reproduced with permission.^[^ ^161^ ^]^ Copyright 2018, American Chemical Society. f) A wearable pacifier based on the chemical‐electrical conversion principle is used to detect glucose content mechanism. Reproduced with permission.^[^ ^168a^ ^]^ Copyright 2019, American Chemical Society. g) An artificial synapse that can accelerate the chemical signal recognition process. i) schematic diagram and ii) the excited response and memory formation behavior under stimulation. Reproduced with permission.^[^ ^169a^ ^]^ Copyright 2021, John Wiley and Sons. h) A schematic diagram of the in vitro taste system with biomimetic artificial taste neurons. Reproduced with permission.^[^ ^169b^ ^]^ Copyright 2022, American Chemical Society.

Sensing units of biomimetic taste sensors commonly use nanomaterials, organic compounds, and biological materials with high sensitivities and selectivities. The taste receptors/cells/tissues in the sensing unit can simulate the sensory mechanism of the human taste system, achieving highly sensitive and selective detection, which is the most commonly used and highly regarded method (**Figure** **13**a–c).^[^ ^158^ ^]^ The key is to maintain the vitalities and stabilities of taste receptors/cells/tissues in vitro. Recently, Wang et al. have committed to developing an in vitro biomimetic taste system.^[^ ^158^, ^159^ ^]^ More recently, they have constructed a taste organoids‐on‐a‐chip system based on the mechanism of endogenous receptor expression in cells.^[^ ^158b^ ^]^ Because of the excellent self‐renewal activities of the taste progenitor cells extracted from C57 mice, the expressive taste receptor cells can retain high cell viabilities to recognize taste stimuli and produce observable changes of extracellular field potentials over seven days, enabling the system to mimic the mammalian taste system to recognize varying degrees of sour, sweet, bitter, and salty tastes. The high biocompatibility indicated by the cell experimental results demonstrates the possibility of system applications in wearable electronics. Contrary to Wang's cell culture conditions, Cho et al. from South Korea were the first to report a flexible biomimetic taste system using an extracellular matrix.^[^ ^160^ ^]^ The biomass microenvironment can maintain the specific phenotypes of primary taste cells in vitro and improve the ability of the system to recognize various tastes.

As wearable biomimetic taste sensors are essentially chemical sensors (real taste perception is the result of the chemical recognition of several characteristic molecules, including nutrients and toxins, in liquid environments by the taste system)^[^ ^161^ ^]^ the primary signal transduction mechanism currently involves the conversion of biochemical signals into electrical signals. The hydrogel‐based flexible electronic tongues developed by Yeom et al.^[^ ^158a^ ^]^ and Lin et al.^[^ ^162^ ^]^ utilized different electrical signals produced by chemical bonding between the target compound and polymer network when in contact to determine the contents of quinine sulfate and polyphenolics, which can reflect bitter and astringent tastes (Figure 13d). Zhao et al.^[^ ^163^ ^]^ and Xue et al.^[^ ^164^ ^]^ synthesized wearable biomimetic taste sensors with high selectivities and abilities to respond to ascorbic acid and alcohol based on coupled enzymatic‐triboelectric and enzymatic‐piezoelectric reactions, respectively. The enzymatic reaction is activated when the target analyte is detected, increasing charge separation and enhancement of the triboelectric/piezoelectric effect, leading to a higher output signal. This approach facilitates indirect detection of the concentration of the target analyte. Ciui et al.^[^ ^161^ ^]^ installed a biomimetic taste perception electronic skin (e‐skin) on a robot finger; it accurately distinguished sweetness, acidity, and spiciness by electrochemically detecting glucose, ascorbic acid, and capsaicin and realized rapid detection of sugar and caffeine in common drinks, providing the possibility for automated taste detection (Figure 13e). By mimicking chemical taste perception, existing studies have also identified lactic acids,^[^ ^165^ ^]^ carbohydrates,^[^ ^165^, ^166^ ^]^ alcohols,^[^ ^167^ ^]^ and biomarkers^[^ ^168^ ^]^ in wearable body fluid sensors (Figure 13f). Additionally, compared with using a microelectrode array as an electron axon to conduct extracellular electrical signals, biomimetic neuromorphic electronics can mimic taste neurons to transmit differential signals of liquids and salts by releasing charge carriers (Figure 13g,h).^[^ ^169^ ^]^

In summary, recognition elements (such as polymer networks, enzymes, taste receptors, and other sensing elements) in biomimetic taste sensors can selectively and sensitively sense target molecules, whereas transducer elements (including transducer electrodes) can convert biochemical signals into electrical signals. Optimized designs of these elements and circuits for signal amplification, filtering, and processing collectively contribute to the development of advanced taste biosensors. Therefore, these elements are critical factors in taste biosensing.

### 4.4 Auditory Perception

Wearable biomimetic auditory sensing is currently of significant importance in the field of sensing as it holds considerable potential to deliver personalized and immersive auditory experiences for users in various domains including entertainment, education, and healthcare. Upon entering mammalian ears, sound waves with different frequencies can trigger vibration of a specific frequency at the cochlear basilar membrane; then, cochlear hair cells transform the vibration into electrical signals and send them to the auditory system to complete auditory perception.^[^ ^22^ ^]^ Therefore, the key to realizing biomimetic auditory sensing and detection functions is to sensitively detect mechanical strain/stress changes caused by sound waves (tiny vibrations).

**Figure 14:**
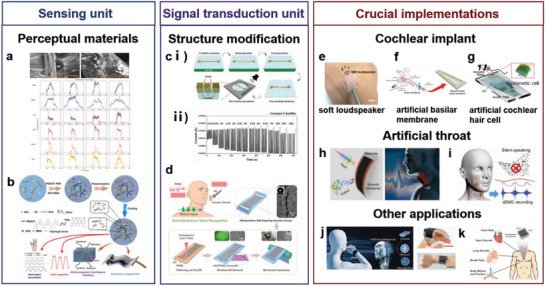
Schematic diagram of biomimetic auditory sensors. a) SEM image of graphene‐based acoustic sensor material and its acoustic application. Reproduced with permission.^[^ ^49^ ^]^ Copyright 2022, Multidisciplinary Digital Publishing Institute, MDPI. b) Schematic illustration of the preparation and acoustic application of the conductive polymer–hydrogel composite. Reproduced with permission.^[^ ^170b^ ^]^ Copyright 2022, Royal Society of Chemistry. c) Cracked structure acoustic sensor. i) fabrication process and ii) the sensitive electrical response to the applied strain. Reproduced with permission.^[^ ^173^ ^]^ Copyright 2020, John Wiley and Sons. d) Self‐cleaning wearable sensor based on the biomimetic jagged microcracks structure thin film, which can be used for anti‐interference speech recognition. Reproduced with permission.^[^ ^174^ ^]^ Copyright 2019, American Chemical Society. e–g) Wearable acoustic sensors as important components of artificial cochlear products, including e) soft microphones. Reproduced with permission.^[^ ^175^ ^]^ Copyright 2018, American Association for the Advancement of Science, AAAS. f) artificial soft substrate membranes. Reproduced with permission.^[^ ^173^ ^]^ Copyright 2020, John Wiley and Sons. g) artificial cochlear hair cells. Reproduced with permission.^[^ ^180^ ^]^ Copyright 2014, John Wiley and Sons. h,i) Wearable acoustic sensors as artificial larynx for voice generation in patients with vocal cord dysfunction, including h) the voice generation patch. Reproduced under terms of the CC‐BY license.^[^ ^181^ ^]^ Copyright 2017, Lu‐Qi Tao et al., published by Springer Nature. i) the tattoo that can recognize voice commands. Reproduced under terms of the CC‐BY license.^[^ ^182^ ^]^ Copyright 2020, Huicong et al., published by Springer Nature. j,k) Other important applications of wearable acoustic sensors, including j) acoustic human–machine interaction. Reproduced with permission.^[^ ^183a^ ^]^ Copyright 2021, John Wiley and Sons. k) disease diagnosis and treatment. Reproduced under terms of the CC‐BY license.^[^ ^186^ ^]^ Copyright 2020, Pranav Gupta et al., published by Springer Nature.

To develop wearable biomimetic auditory sensors, identifying suitable sensing materials for the sensing unit is essential. An ideal sensing material should demonstrate high sensitivity and selectivity to specific sound frequencies and excellent mechanical properties and biocompatibility. In recent years, several materials, including CNTs, graphene, polymeric composites, and piezoelectric materials, have been explored for use in biomimetic auditory sensors (**Figure** **14**a,b).^[^ ^49^, ^170^ ^]^ Among these materials, piezoelectric materials have gained significant attention due to their unique abilities to convert mechanical energy into electrical energy, rendering them an ideal choice for the sensing unit of biomimetic auditory sensors.^[^ ^171^ ^]^

Mechanism of signal transduction in wearable auditory sensing devices is based on the transformation of mechanical signals into electrical signals, thus emphasizing the crucial role of material structures with extensive contact areas in enhancing the sensitivity of signal transmission. Depending on its susceptible biomimetic hierarchical structure, a wearable AuNW/PDMS ultrathin hierarchical sheet can detect small vibrations from music (<13 Pa) to sense pressure changes.^[^ ^172^ ^]^ A cracked structure was employed in biomimetic film development, and a wearable artificial basilar membrane (ABM) with high‐frequency selectivity (319–1951 Hz) (Figure 14c) was successfully fabricated.^[^ ^173^ ^]^ Suitable collaboration of these two biomimetic structures can also endow the acoustic sensor with self‐cleaning ability (Figure 14d).^[^ ^174^ ^]^ A wearable soft loudspeaker with an ultrathin micropyramid structure exhibited significant triboelectric effect. The average reliability of a personal voice security system developed based on this loudspeaker reaches 98.6 ± 0.8%, which is similar to that of other commercial products (99.1 ± 0.6%).^[^ ^175^ ^]^ Another micropyramid structure employed as a flexible electronic eardrum can achieve a frequency domain of 20–13 000 Hz, which is similar to that of the human ear.^[^ ^176^ ^]^ Additionally, wearable TENGs and self‐powered acoustic sensors based on the biomimetic auditory sensing principle are being widely developed because acoustic sensing is often accompanied by electrostatic induction and contact electrical coupling effects.^[^ ^177^ ^]^

As the most important application of wearable biomimetic auditory sensors, emerging high‐performance cochlear implant products are promising alternatives to high‐risk surgical treatments, helping patients with hearing impairments to obtain sound information.^[^ ^173^ ^]^ Although traditional cochlear implant conceptual products, such as hearing aids, have been extensively popularized in this century, they demonstrate some shortcomings, for example, rigid materials cannot fit the human skin and affect the comfort of wearing and hearing correction effect is little. Therefore, a new generation of flexible wearable cochlear implant products has attracted wide attention in recent years. The soft loudspeaker mentioned earlier can be used as an important component of new‐generation wearable cochlear implants (Figure 14e).^[^ ^175^ ^]^ Moreover, lighter and softer wearable flexible ABMs have recently been constructed and are expected to replace the rigid basilar membranes in traditional cochlear implants (Figure 14f). As mentioned above, sound waves can produce signals by triggering vibrations in mammalian eardrums; in addition to the common triboelectric‐type materials,^[^ ^178^ ^]^ flexible ABMs can use piezoelectric,^[^ ^179^ ^]^ resistive,^[^ ^173^ ^]^ and capacitive^[^ ^22^ ^]^ materials to mimic cochlear tonotopy and realize acoustic‐to‐electric signal conversion. Jang et al.^[^ ^178^ ^]^ developed a triboelectric ABM with frequency selection ranging from 294.8 to 2311 Hz. A piezoelectric ABM using a similar isosceles trapezoid geometry structure exhibits a frequency selection range of 3–8 kHz, which is closer to the most sensitive stimulation range of the human cochlea,^[^ ^179a^ ^]^ and another piezoelectric ABM demonstrates a frequency selection range similar to that of the human ear (2.92–12.6 kHz).^[^ ^179b^ ^]^ Additionally, artificial biomimetic cochlear hair cells are an important form of wearable biomimetic auditory sensors (Figure 14g).^[^ ^180^ ^]^

In addition to helping the deaf and hard of hearing regain their hearing sense, auditory bionics are crucial for helping people who have lost voice (Figure 14h,i). For example, Ren et al. fabricated a wearable artificial throat that integrates the function of generating and detecting sound, helping mute persons to “speak” by detecting their tiny throat vibrations.^[^ ^181^ ^]^ Tattoo‐like patches, proposed by Liu et al., can provide instruction recognition with 92.33% accuracy for patients with voice loss by detecting surface electromyography.^[^ ^182^ ^]^ Moreover, highly conformal wearable flexible devices have gradually become attractive for speech recognition. Therefore, these devices can be combined with artificial intelligence to offer wearable acoustic HMI and robot‐intelligent hearing platforms, strengthen human–machine acoustic interaction, and realize high‐performance speech recognition (Figure 14j).^[^ ^183^ ^]^ The mechanical‐acoustic dynamics represented by mechanical waves are closely related to many natural physiological activities in the human body; thus, wearable biomimetic auditory devices can realize medical monitoring, such as cardiovascular disease diagnosis^[^ ^184^ ^]^ and intestinal monitoring^[^ ^185^ ^]^ in combination with medical methods including ultrasound (Figure 14k).^[^ ^186^ ^]^

### 4.5 Visual Perception

As the most important sensory function of most organisms (such as humans and insects), visual perception accounts for 80% of external information acquisition.^[^ ^22^ ^]^ Therefore, mimicking the powerful visual perception function of organisms is significant for medical care, human–machine interaction, and public safety. Presently, the primary wearable sensors based on visual bionics are artificial visual systems including biomimetic eyes. However, all wearable artificial vision system products that mimic human eyes or insect compound eyes face many challenges such as visual field range, device flexibility, perceptual sensitivity, and imaging clarity.^[^ ^187^ ^]^

**Figure 15:**
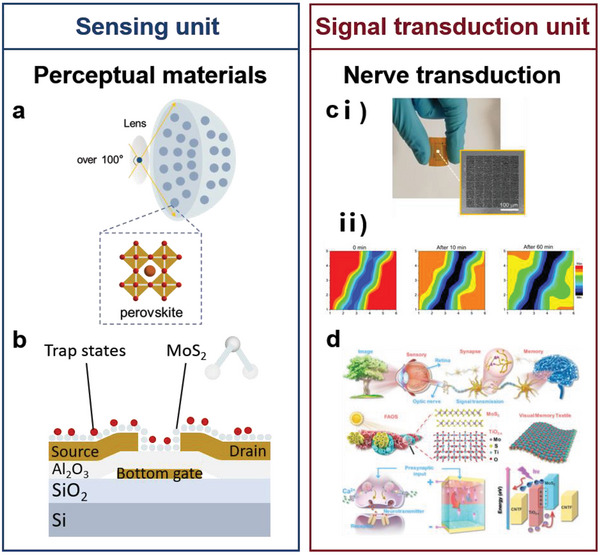
Schematic diagram of biomimetic visual sensors. a) Working mechanism and crystal structure of the materials in the biomimetic eyes based on perovskite NWs. b) Schematic diagram of the MoS_2_ phototransistor. c) Quasi‐2D electron gases artificial synaptic visual system, including i) the appearance and ii) the imaging and memory behavior of the artificial visual system after different memory times. Reproduced under terms of the CC‐BY license.^[^ ^192^ ^]^ Copyright 2020, You Meng et al., published by American Association for the Advancement of Science, AAAS. d) Schematic diagram of the human visual perception and memory system and the fiber‐like artificial optoelectronic synapse that can be integrated into fabrics. Reproduced with permission.^[^ ^193^ ^]^ Copyright 2023, Elsevier.

Recently, researchers have made many effective attempts to overcome these challenges. For example, high‐quality imaging has been realized using an ultrathin molybdenum disulfide (MoS_2_)‐based flexible optoelectronic device,^[^ ^188^ ^]^ which can be combined with neural interfacing electrodes to form implantable artificial vision systems. The soft device can protect the rat retina and stimulate local nerves in the membrane to collaboratively respond to external optical signals simultaneously. To achieve flexible biomimetic visual imaging, the widest field of vision is also an important factor for further improving the performances of wearable artificial vision systems. The traditional 2D planar structure limits the detection abilities of sensors at large angles. A flexible curved surface imager developed on a kirigami structure maintained a resolution of 32 × 32 pixels when subjected to 30% biaxial strain, achieving compatibility between the detection of a curved field view and high‐quality imaging.^[^ ^189^ ^]^ A hemispherical retina based on high‐density perovskite nanomaterials can reach a diagonal visual field of over 100°; nevertheless, planar structures cannot exceed a diagonal visual field of 70°. By further optimizing the pixel distribution, its field of view reached 130°, close to the human field of view (150–160°) (**Figure** **15**a).^[^ ^190^ ^]^ Lee et al.^[^ ^24^ ^]^ synthesized a flexible photoelectric sensor array with a biologically inspired comb‐shaped structure. When attached to a spherical substrate, it can achieve panoramic vision, break through hemispherical vision (approximately 180°), and exhibit the same visual perception underwater and potential to be used in wearable electronics.

Visual biomimetic technology is closely related to the principle of optical imaging. Adjusting the relationship between sensors (materials) and light is also important. Liao et al.^[^ ^191^ ^]^ introduced a charge‐trap state into MoS_2_, resulting in strong optical dynamic response characteristics of the material (Figure 15b). A visual biomimetic sensor prepared based on this material can adapt to light and dark environments and recognize images; therefore, it demonstrates a wide perception range of up to 199 dB. By combining CNTs and perovskite quantum dots, Zhu et al.^[^ ^187^ ^]^ successfully developed a flexible vision sensor with a light response value of 5.1 × 10^7^ A W^−1^ and specific detectivity of 2 × 10^16^ Jones, which can perform intelligent image recognition under low light. A new generation of wearable neuromorphic biomimetic visual devices has also attracted our attention. Meng et al.^[^ ^192^ ^]^ constructed an artificial synaptic visual system using quasi‐2D electron gases at the interface of superlattice NWs. Owing to the excellent ion transport efficiency of the NW network, artificial synapses of quasi‐2D electron gases consume nearly as much energy in signal transduction as that consumed by the organism; simultaneously, the sensor exhibits the characteristics of high‐performance light detection, brain‐like information processing, and memory ability (Figure 15c). Another fiber‐shaped artificial optoelectronic synapse can mimic the electrical and light‐induced functions of biological synapses, such as memory, learning, and excitement, and can be integrated into wearable textiles to develop a new generation of wearable visual devices (Figure 15d).^[^ ^193^ ^]^ Wilson et al.^[^ ^194^ ^]^ also implanted transparent microelectrodes in the retrosplenial cortex of adult mice to realize the monitoring of brain‐like neural visual signals.

### 4.6 Multisensory Perception

Inspired by the concept of sensor arrays, researchers have developed a wearable e‐skin integrated with a sensor array using advanced microelectronics technology and solved the problems, such as thickness,^[^ ^195^ ^]^ insufficient flexibility,^[^ ^196^ ^]^ signal interference,^[^ ^197^ ^]^ cumbersome detection circuit,^[^ ^198^ ^]^ and insufficient signal processing ability.^[^ ^199^ ^]^ caused by integrated multi‐sensors. The current high‐performance multiarray e‐skin can further mimic the ability of human skin to perceive multiple stimuli, which is of considerable significance for the synchronous detection of multiple complex stimuli from the skin to achieve accurate and selective diagnosis of various diseases.^[^ ^200^ ^]^ Accordingly, by further deepening the functions, researchers have gradually realized the integration of several biomimetic senses in a single wearable electronic sensing system and have contributed to the advancement of artificial intelligence and smart Internet of Things systems.

**Figure 16:**
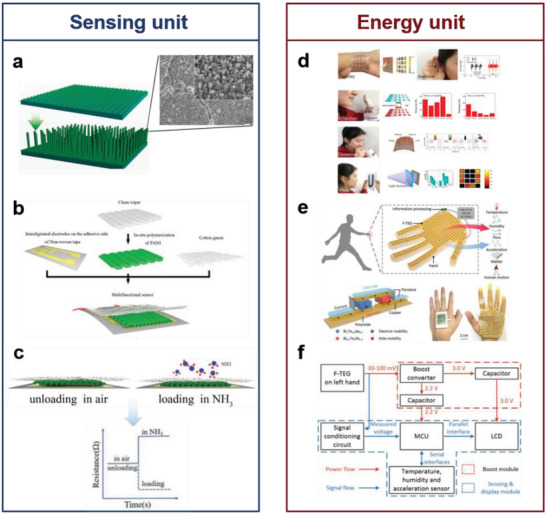
Schematic diagram of biomimetic multisensory sensors. a) Fabrication process and SEM images of the PANI NW arrays on a PDMS substrate for the visual‐olfactory sensor based on the biomimetic snake scale structure. Reproduced under terms of the CC‐BY license.^[^ ^201a^ ^]^ Copyright 2017, Guofa Cai et al., published by Springer Nature. b,c) The fabrication process and schematic illustrations of the gas‐ and pressure‐sensing mechanisms and resistance changes in response to different stimuli of PANI@textile‐based multisensory sensor. Reproduced with permission.^[^ ^201b^ ^]^ Copyright 2018, John Wiley and Sons. d) Biomimetic sensory sensor based on the frictional electrification principle that can mimic the five senses. Reproduced with permission.^[^ ^203^ ^]^ Copyright 2018, Elsevier. e,f) Schematic diagram and working principle of the self‐powered multi‐sensory e‐skin system based on a thermoelectric generator. Reproduced with permission.^[^ ^204^ ^]^ Copyright 2020, John Wiley and Sons.

A key challenge in developing wearable multibioinspired sensory systems is to achieve highly sensitive and selective sensory responses. Bioinspired materials and conductive polymers, such as polyaniline (PANI), are commonly used to address this issue. These materials can mimic the structures and functionalities of biological sensory organs, enabling them to respond to various unrelated stimuli, and exhibit excellent wearabilities and biocompatibilities.^[^ ^201^ ^]^ As a unique conducting polymer, PANI electrochemically responds to gas, temperature, humidity, and strain.^[^ ^202^ ^]^ It is considered a natural multisensory sensing‐based conductive material; however, its chain rigidity limits its applications in flexible wearable devices. Therefore, Cai et al.^[^ ^201a^ ^]^ grew a PANI material with a planar particulate film and vertically aligned NWs on a soft PDMS substrate to form a snake scale‐like stretchable structure, which solved the problems of flexibility and stretchability and possessed tactile and olfactory perceptions similar to those of snakes (**Figure** **16**a). Its highest gauge factor reached 149, and it specifically recognized methane. Tang et al.^[^ ^201b^ ^]^ reported a low‐cost preparation method for a wearable conductive material by in situ polymerization of PANI on a flexible wiper. The resulting product responded to a wide range of pressures (300 Pa–30 kPa) and an excellent linear response to NH_3_ (Figure 16b,c). Using similar multilayered structures, wearable multimodal sensory e‐skins have been developed that can detect multidirectional tactile motions (e.g., shear, pinch, and spread) and simultaneously analyze thermal information without signal interference to form a special sensation and thermoperception.^[^ ^201c^ ^]^

High energy consumption of multisensor arrays in wearable biomimetic sensing systems is also a challenge. Traditional battery‐powered systems demonstrate limitations including low energy densities, short lifespans, and long charging times. Self‐powered technologies offer benefits such as low maintenance requirements, extended lifetimes, and energy efficiencies. Typical self‐powered principles include environmental, mechanical, and biological energy conversion. By coupling the triboelectric and charge effects, a self‐powered multiperception wearable electronic skin can autonomously and continuously complete the entire process from information reception to processing by relying on the energy supply of the wearer's motion capacity without an external power supply. It can simulate the five senses (tactility, audition, olfaction, gustation, and vision) of human beings and complete actual behaviors, for example, sensing pressure, hearing words, smelling smells, tasting drinks, and recognizing pictures (Figure 16d).^[^ ^203^ ^]^ The e‐skin developed by Yuan et al. using a thermoelectric generator as the core can capture energy from the wearer's body heat and complete analog perception functions such as thermoperception and tactile perception (Figure 16e,f).^[^ ^204^ ^]^ With the advancement of technology, self‐powered solutions will be increasingly integrated into wearable biomimetic multisensory systems, providing a reliable and sustainable source of power.

## 5 A Path Forward for Biomimetic Wearable Sensing Systems That Incorporate the Required and Advanced Technologies

### 5.1 Combination with Artificial Intelligence

With improvements in biomimetic wearable sensing system performance, traditional data processing paradigms, including threshold limits and basic 1D mathematical models, have demonstrated a lack of processing ability when faced with different patterns of data from the sensor arrays.^[^ ^205^ ^]^ As a momentous technology in the field of artificial intelligence,^[^ ^206^ ^]^ the introduction of machine learning algorithms with multidimensional and multimodule paradigms can effectively improve the computational ability of the data processing module by maintaining the original hardware design of the sensing system and further promote the manufacturing of advanced intelligent wearable sensors.^[^ ^207^ ^]^ Additionally, biomimetic wearable sensing systems can be used in the development of other technologies (including intelligent sensing robot arms/hands and HMI) in the field of artificial intelligence, which is conducive to expanding the application prospects of these systems and can promote deeper interactions between people and virtual worlds.

#### 5.1.1 Introducing Machine Learning Algorithm

In the field of wearable sensing electronics, the intervention of biomimetic technology and machine learning algorithms can be regarded as two ways to improve the sensing performance. However, they are complementary and mutually promoting relationship, which is reflected in the following two aspects.

**Figure 17:**
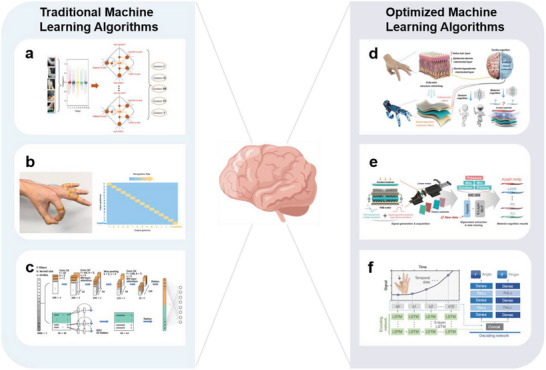
Schematic diagram of biomimetic wearable sensors with integrated machine learning algorithms. a) Schematic diagram of a biomimetic wearable sensor for rapid gesture recognition using long short‐term memory algorithm. Reproduced with permission.^[^ ^209^ ^]^ Copyright 2020, Elsevier. b) Wearable sensor worn on fingers for sign language recognition and results of support vector machine (SVM) classification. Reproduced with permission.^[^ ^210^ ^]^ Copyright 2021, Elsevier. c) Working principle of a robotic hand utilizing piezoelectric signals and machine learning techniques for softness classification of objects. Reproduced under terms of the CC‐BY license.^[^ ^212^ ^]^ Copyright 2022, Ye Qiu et al., published by Springer Nature. d) Schematic diagram of an artificial tactile cognition system based on artificial neural network and e) Flowchart of neural network‐assisted material cognition process in artificial tactile cognition system, including data generation, acquisition, training, and actual cognition processes. Reproduced with permission.^[^ ^213^ ^]^ Copyright 2022, John Wiley and Sons. f) Schematic diagram of optimizing hand motion measurement space with long short‐term memory algorithm and Dropout in artificial neural network encoding. Reproduced under terms of the CC‐BY license.^[^ ^215a^ ^]^ Copyright 2020, Kyun Kyu Kim et al., published by Springer Nature.

On the one hand, in the face of a large amount of human physiological data, such as biomarkers, biological potentials, and physical signals, obtained by high‐performance biomimetic wearable sensors, traditional data processing technology cannot play an effective role because of the limitations of capacity, classification efficiency, and feature extraction technology.^[^ ^205^ ^]^ With the arrival of the big data era, machine learning algorithms can be used as terminal information processing methods to assist wearable devices in achieving high‐efficiency and performance‐sensing detection and target recognition. This advantage is reflected in the data processing speed. The e‐skin developed by Yao et al. by constructing micropyramidal biomimetic conductive polymer morphologies on flexible substrates exhibited high‐pressure sensitivities (> 10^7^Ω kPa^−1^). By introducing a machine learning algorithm for high‐speed data processing, this e‐skin can simultaneously recognize and integrate stress information from different directions and evolve from ordinary pressure recognition to realizing fast surface texture recognition of objects.^[^ ^208^ ^]^ Based on the biomimetic porous fiber structure and field‐effect synaptic transistor amplifying the electrical signals generated by the frictional effect, Miao et al.^[^ ^209^ ^]^ and Shan et al.^[^ ^210^ ^]^ realized fast gesture recognition using machine learning algorithms (**Figure** **17**a,b). Nevertheless, the advantage lies in its capacity for data processing. A novel type of e‐skin based on an asynchronously coded neuromimetic architecture instead of the previously inefficient serial information reading way simultaneously realized high‐speed analysis of thermotactile information from 10 000 sensors.^[^ ^211^ ^]^ Qiu et al. used a machine learning algorithm to endow a multisensory e‐skin with the ability to recognize the softnesses of objects via multisignal synthesis, thus providing intelligent sorting ability to a robot hand installed with e‐skin (Figure 17c).^[^ ^212^ ^]^

On the other hand, biomimetic technology can optimize the traditional machine learning algorithm architecture. A representative example is a biomimetic machine‐learning algorithm inspired by the neural network of an animal brain, called an artificial neural network. Compared with traditional machine learning algorithms, artificial neural networks can automatically learn data features via multilayer neural network structures and demonstrate stronger parallel computing capabilities for processing unstructured data, which accelerate the training and prediction processes. Therefore, it has become a suitable intelligent algorithm for processing the information set collected by wearable devices and has rapidly developed in the past decade.^[^ ^205^ ^]^ By mimicking the structures of human vellus hair, Niu et al. developed an e‐skin that achieved an ultrahigh sensitivity of 8053.1 kPa^−1^ (<1 kPa) and a linear sensitivity of 3103.5 kPa^−1^. On this excellent basis, they introduced the neural network as “brain,” further constructed a gesture morphology cognition glove‐mounted system and an intelligent material cognition system, and realized the leap from tactile perception to tactile cognition (Figure 17d,e).^[^ ^213^ ^]^ Sundaram et al.^[^ ^214^ ^]^ employed deep convolutional neural networks to successfully realize intelligent real‐time cognition of surface features, weight, and other information of the captured objects (26 in total). Furthermore, convolutional neural networks have successfully assisted the single‐sensor replacement of arrays for finger motion detection and analyzed visual‐tactile fusion data to guide robots to move forward with an error rate of less than 4% (Figure 17f).^[^ ^215^ ^]^

#### 5.1.2 Developing Intelligent Sensing Robot Arms/Hands

**Figure 18:**
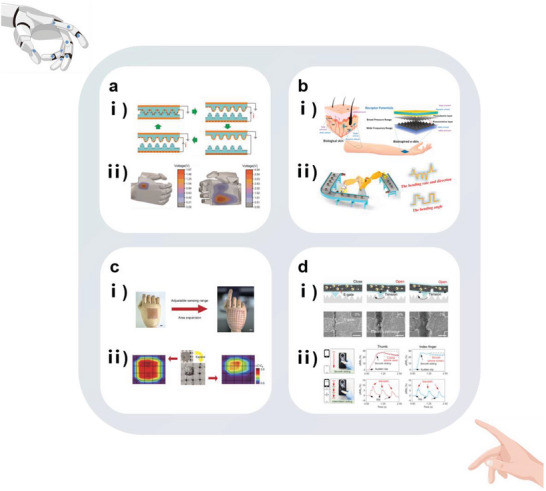
Intelligent robotic arms/hands based on biomimetic wearable sensors. a) Electronic skin with plant‐inspired interlocking microstructures and its integration with a robotic hand. i) Schematic diagram of normal force sensing principle and ii) voltage profile of handshake between robotic arm and human. Reproduced with permission.^[^ ^216^ ^]^ Copyright 2019, John Wiley and Sons. b) Pressure sensor based on biomimetic skin interlocking structure. i) Structural diagram and ii) schematic diagram of intelligent robotic hand equipped with the sensor for grabbing and transporting delicate objects on assembly line. Reproduced with permission.^[^ ^217^ ^]^ Copyright 2020, Elsevier. c) skin‐inspired flexible sensing matrix network as artificial tactile skin for hand perception and regulation. i) Scalability concept and ii) position detection of pressure load even with 300% expansion. Reproduced under terms of the CC‐BY license.^[^ ^218^ ^]^ Copyright 2018, Qilin Hua et al., published by Springer Nature. d) Robot electronic skin with mechanical gate‐controlled electronic channel. i) Sliding sensing mechanism of R‐skin and ii) detection of sliding smartphone status. Reproduced under terms of the CC‐BY license.^[^ ^219^ ^]^ Copyright 2022, Sheng Li et al., published by American Association for the Advancement of Science, AAAS. The “mechanical hand” and “human hand” elements in the picture were created by figdraw.

A high‐performance biomimetic wearable robot e‐skin can offer high recognition accuracy, diverse recognition methods, and detection scenes and further assist robot arms/hands in completing the perception, recognition, and grasping functions, increasing the possibility of its modern intelligent application, which is of considerable significance for the development of intelligent robots and prostheses. In recent years, interlocked microstructured e‐skins inspired by human skin and plants have been extensively used in the synthesis of intelligent robotic arms due to their sensitivities to signal transmission. The TENG developed by Yao et al.^[^ ^216^ ^]^ by incorporating polytetrafluoroethylene tinny burrs into tribolayers to mimic the interlocked microstructure of a plant can effectively improve the pressure sensitivity by 14‐fold; thus, the robot arm wearing this e‐skin can examine the pressure and even the angle when touching objects and then perceive the handshaking gestures and roughnesses of objects (**Figure** **18**a). Qiu et al. fabricated an e‐skin by adopting dual‐mode interlocked piezoelectric and piezoresistive materials to cooperatively sense pressure. When applied to an intelligent robot hand, the manipulator can intelligently sense and sort items on the assembly line (Figure 18b).^[^ ^217^ ^]^ Additionally, a skin‐inspired flexible sensor matrix network consisting of 100 sensor nodes simultaneously and undifferentiably detected temperature, strain, humidity, UV, magnetic field, and pressure signals. More importantly, it demonstrated compatibility with smart prosthetics. Even when the expansion was 300% or the sensing area expansion was 25 times, it retained excellent weight perception and object recognition abilities (Figure 18c).^[^ ^218^ ^]^

In addition to the abovementioned static tactile recognition, the dynamic tactile recognition abilities of intelligent robot arms, which are considered important parts of improving the intelligence of robot arms, benefit from the development of an e‐skin inspired by this skin‐inspired interlocked structure. Shao et al. reported a robotic e‐skin with mechanically gated electron channels.^[^ ^219^ ^]^ The rough fingerprint structure on its surface can amplify and transmit mechanical stimuli under stress. Because stimuli are internally transmitted, this e‐skin can sensitively control the opening and closing of electron gates and reflect the electric flow via them as electrical signals. Therefore, a robot arm based on this e‐skin can quickly identify the texture features of micron‐level structure with response frequency exceeding those of humans (485 Hz). More importantly, it can realize sliding sensing functions, such as the detection of the sliding state and real‐time feedback capture, similar to those of humans (Figure 18d). Similarly, by mimicking the rough texture of a human fingerprint, another robot e‐skin has been constructed that can recognize the detailed textures of static objects and identify the shear force generated during the object dragging process by calculating the maximum triboelectric voltage/piezoelectric voltage and the sum of the amplitude of the fast Fourier transform frequency.^[^ ^126^ ^]^ Bao et al.^[^ ^220^ ^]^ adopted a similar structure but introduced a pyramid interlocked microstructure to further strengthen the triboelectric effect for distinguishing normal and tangential forces during sliding.

#### 5.1.3 Promoting HMI

Over the past few decades, HMI has made substantial progress in communication and education; however, its applications are limited by inferior signal acquisition.^[^ ^221^ ^]^ Thus, high‐performance biomimetic wearable sensors can effectively improve the dimensions and quality of signal acquisition, quickly capture the wearer or environmental information, and provide more diverse information feedback channels and are expected to improve the level of medical care and intelligent technology.

**Figure 19:**
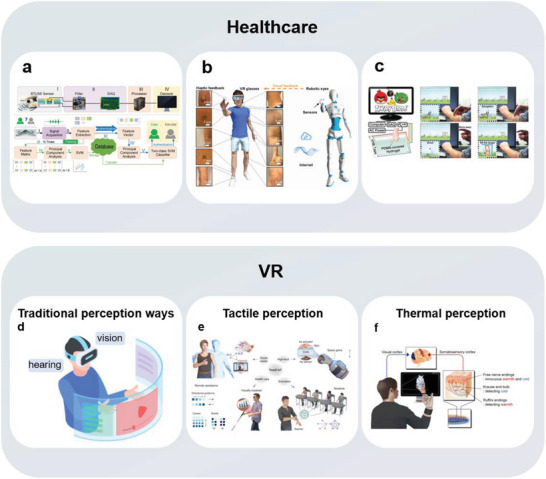
Schematic diagram of biomimetic wearable sensors in human‐machine interaction applications. a) Schematic diagram of a communication system for people with disabilities based on detecting micro movements of the masseter muscle. Reproduced under terms of the CC‐BY license.^[^ ^221^ ^]^ Copyright 2021, Hong Zhou et al., published by John Wiley and Sons. b) A man wearing a closed‐loop HMI medical electronic skin remotely controls a robot through tactile and visual feedback via the Internet, demonstrating the possibility of remote care. Reproduced under terms of the CC‐BY license.^[^ ^222^ ^]^ Copyright 2022, Yiming Liu et al., published by American Association for the Advancement of Science, AAAS. c) Operation of a wearable HMI touch pad based on hydrogel. Reproduced with permission.^[^ ^224^ ^]^ Copyright 2020, John Wiley and Sons. d) Schematic diagram of traditional VR/AR devices’ perception ways. e) Possible application of wireless tactile sensing‐based touch internet of things in emerging fields of social media in the future. Reproduced under terms of the CC‐BY license.^[^ ^227^ ^]^ Copyright 2022, Dengfeng Li et al., published by American Association for the Advancement of Science, AAAS. f) Schematic diagram of a VR smart glove providing thermal feedback. Reproduced under terms of the CC‐BY license.^[^ ^230b^ ^]^ Copyright 2020, Seung‐Won Kim et al., published by Springer Nature.

As the medical industry has suffered from the coronavirus pandemic in recent years, the development of intelligent medical devices that can be used at a safe distance from the suspected patients and integrate detection and diagnosis functions is of considerable significance for the protection of medical workers and patients. Accurate and rapid acquisition of physiological signals using wearable sensors is essential for safe and precise monitoring in medical human–machine interaction technology, which requires a carefully selected high‐level signal transduction design and sensor array. Based on a mechanism similar to that by which internal airflow triggers external vocal vibrations and produces a voice when frogs sing, Zhou et al.^[^ ^221^ ^]^ used soft PDMS elastic membranes to amplify the masseter muscle's real‐time micromovements in the subject, converted mechanical signals into visual electrical signals, introduced a communication protocol (Morse code) and machine learning algorithms into HMI, and successfully developed a communication system appropriate for disabled persons/patients (**Figure** **19**a). Liu et al.^[^ ^222^ ^]^ proposed a closed‐loop HMI medical system compatible with the whole body, which was expected to realize the remote care of patients with infectious diseases via the integration of various biomimetic sensory functions and communication means (Figure 19b). The hydrogel‐based flexible e‐skin developed by Park et al.^[^ ^223^ ^]^ using a biomimetic skin structure and numerous microphone arrays can exhibit large‐area tactile perception and provide whole‐body human–computer interaction. Another novel hydrogel‐based human‐machine interactive touch pad is also promising to offer remote communication between patients and doctors (Figure 19c).^[^ ^224^ ^]^

VR and AR are widely applied as essential product forms of human‐machine interaction interfaces. A promising way to achieve highly immersive VR and AR applications is to introduce different biomimetic sensory experiences into VR/AR devices to enhance the degree of human‐machine interactions. Existing VR/AR devices primarily create immersive senses by establishing visual and auditory experiences (Figure 19d).^[^ ^225^ ^]^ Although the degree and dimensions of simulation of these two senses are constantly improving, the absence of other senses still prevents us from experiencing the real world. Fortunately, tactile feedback is now a reality in human–machine interaction interfaces, affording diverse tactile information, including texture and motion behavior, via mechanical signals.^[^ ^226^ ^]^ For example, the e‐skin fabricated by Li et al.^[^ ^227^ ^]^ demonstrates dual functions of tactile perception according to the principle of the generator and tactile feedback based on the principle of mechanical vibration; thus, both parties wearing the e‐skin can feel tactile sensation of each other via wireless signal transmission, achieve surprising non‐contact remote tactile communication in the form of a touch intercom, and fill the gap between wireless tactile experience and VR/AR (Figure 19e). Shi et al.^[^ ^228^ ^]^ and Yu et al.^[^ ^229^ ^]^ reproduced high‐level tactile information, such as positions, motion trajectories, surface features, and weights of perceived objects, on the skin surface using electrostatic discharge effects and millimeter‐scale vibrators. This is a significant advancement as compared to the vague tactile information related to force. In addition to the implementation of haptic feedback, some studies have simultaneously introduced tactile and thermal perception into VR devices (Figure 19f).^[^ ^230^ ^]^ Moreover, the introduction of smell can enhance the wearer's experience such as the experience of searching for gas sources.^[^ ^231^ ^]^

### 5.2 Combination with Synthetic Biology

**Figure 20:**
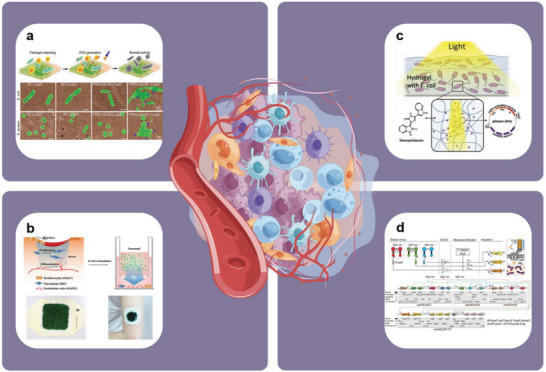
The combination of biomimetic wearable sensors and synthetic biology. a) Schematic diagram and bactericidal effect (blue arrows indicating damaged area of bacterial cells) of engineered living material for skin wound repair based on photosensitive bacterial cellulose releasing reactive oxygen . Reproduced with permission.^[^ ^232a^ ^]^ Copyright 2022, John Wiley and Sons. b) Principle and appearance of microalgae gel skin patch for dynamic delivery of dissolved oxygen. Reproduced under terms of the CC‐BY license.^[^ ^235^ ^]^ Copyright 2020, Huanhuan Chen et al., published by American Association for the Advancement of Science, AAAS. c) Schematic diagram of light‐controlled drug delivery function of antitumoral drug deoxyviolacein achieved by photo‐controllable biocompatible hydrogel material. Reproduced with permission.^[^ ^236^ ^]^ Copyright 2018, John Wiley and Sons. d) Translation of schematic diagram of optogenetically controlled bio‐coating. Reproduced with permission.^[^ ^241^ ^]^ Copyright 2019, John Wiley and Sons. The central cell pattern and the purple box background in the picture were created by figdraw.

Conventional wearable sensing systems contain multiple modules. Sensing requires many steps including signal transduction and data analysis, rendering the entire behavioral process more complicated. For application of these systems in medical diagnosis, a rapid, accurate, and simple perception‐response process is highly preferred (**Figure** **20**a).^[^ ^232^ ^]^ In nature, biological cells comprise attractive functions and internal behavioral logic. Living cells have a natural perception advantage for biological signals, such as bacteria and toxins, over most inorganic materials, which is reflected in their dynamic reactions and sensitivities.^[^ ^233^ ^]^

Advancement of synthetic biology has enabled researchers to facilitate heterologous expressions of biological components, for instance, cells, tissues, and biomolecules, at inorganic interfaces. However, when live bacteria/biomolecules are used in wearable devices, fully considering their safety and biocompatibility is crucial because these devices need to be attached to human skin. In this regard, the safeties of both the substrate and bacterial strains used are of utmost importance. Lina et al.^[^ ^234^ ^]^ demonstrated a hydrogel‐based wearable skin patch loaded with *Bacillus subtilis* spores, which not only exhibited a high degree of biocompatibility with the skin, but also specifically detected and killed *Staphylococcus aureus* in skin wounds. Another nontoxic skin patch encapsulated with cyanobacteria designed by Chen et al. does not cause an immune response and can employ living bacteria to spontaneously sense and dynamically deliver dissolved oxygen to the wound (Figure 20b).^[^ ^235^ ^]^ A hydrogel composite based on an active endotoxin‐free *Escherichia coli* strain can induce bacteria to secrete the antitumor drug deoxyviolacein in a photoregulated manner, realizing wearable drug delivery (Figure 20c).^[^ ^236^ ^]^

Sensing components of biohybrid wearable sensors include live cells, extracellular systems (including enzymes, antibodies, and cytoskeletons), and biological macromolecules (such as nucleic acids and peptides).^[^ ^237^ ^]^ Compared to live cells, extracellular systems and biological macromolecules offer advantages including the ease of large‐scale production and modification, enabling optimization during design and preparation to achieve higher sensitivity, specificity, and stability for use in bioreactors, detection, and molecular recognition in sensors.^[^ ^157^, ^238^ ^]^ Nevertheless, extracellular systems and biological macromolecules lack self‐replication and regeneration abilities, requiring regular replacement. Moreover, their sensitivities and specificities may be affected by environmental factors (for example, temperature and pH), and the traditional production and purification methods for biological macromolecules are expensive, limiting their application range.^[^ ^239^ ^]^ Therefore, the selection of sensing components should be based on the specific application requirements and should be comprehensively considered.

Nowadays, biological genetic inheritance/synthetic pathways have become an important means of optimizing the internal circuitries of inorganic sensing platforms.[^[^ ^240^ ^]^ A secretion system based on three variants of curling, modulated by red, green, and blue light, can be used to prepare high‐resolution biological coatings on cotton surfaces, providing new ideas and pathways for circuit optimization (Figure 20d).^[^ ^241^ ^]^ These biological coatings can transmit the functions (such as toxin detection and odor release) of living organisms to wearable devices or clothing, thereby facilitating the development of biocircuit‐based intelligent clothing.^[^ ^242^ ^]^ Additionally, using hydrogen peroxide regulated by oxygen reduction, gene‐circuit engineered bacteria can interpret and translate electronic signals and offer feedback, thus realizing an autonomous electronic biological platform.^[^ ^243^ ^]^ This biocircuit platform can be employed in biological sensors, computers, and memory. These examples demonstrate the significant progress made in optimizing the circuitries of inorganic sensing platforms by biological genetic inheritance/synthetic pathways, thereby providing new ideas and pathways for circuit optimization.

## 6 Challenges and Outlook

As research on biomimetic wearable electronic sensors has deepened, the focus of research in recent years can be summarized as a shift from static to dynamic, single‐dimensional to multidimensional, individual to array, and traditional electronics to interdisciplinary integration. These changes render the new generation of flexible sensing devices more portable, with richer types of sensing information,^[^ ^79^, ^195^, ^203^ ^]^ more sensitive information monitoring,^[^ ^130^, ^137^ ^]^ more diverse monitoring scenarios,^[^ ^13^, ^60^, ^244^ ^]^ and smaller overall system sizes.^[^ ^27^, ^50^, ^200^ ^]^ These developments bring us closer to portable active medical sensing^[^ ^2^, ^3^, ^7^ ^]^ and have been extensively used to assist robot arms to perceive external information^[^ ^216^, ^217^, ^218^ ^]^ and construct human‐machine interaction interface.^[^ ^221^, ^222^, ^223^ ^]^

### 6.1 Challenges

Development of high‐performance biomimetic wearable sensing systems faces several critical challenges including material design and selection, integration capability and stability of the system, attainment of advanced sensing capabilities, and integration of machine learning and synthetic biology technologies, increasing the intricacy of the development process. To overcome these challenges, a multidisciplinary approach that utilizes diverse knowledge domains, such as materials science, engineering, and computer science, is required. This section explores these pivotal challenges and offers possible solutions.

#### 6.1.1 Challenges in Materials Design

Development of materials is of significant importance for enhancing the performance of the system; however, some challenges remain to be addressed, which can be divided into three aspects. First, further research is needed to establish rational design strategies for engineering materials to fit system requirements, and the core is to define the sequence–structure–function–property relationships of engineered materials. As abovementioned, during material design and testing, the correct sequence and structure designs are beneficial for realizing functions and satisfaction of performance, revealing the progressive relationship between sequence, structure, function, and property; nevertheless, accurately performing their connection via design is difficult. Many functional structures derived from different biomimetic models have little difference in the actual target performance index, which renders the performance improvement challenging. Therefore, the multi‐aspect influences of the sequence and structure of wearable engineering materials on their functions and performance must be clearly determined to provide this logical chain a positive stimulus and apply it to the synthesis of wearable engineering materials.

Second, compared with the previous static research, dynamic “smart” materials (materials dynamically responsive to light, magnetic field, electric field, and temperature) are more attractive because of their mobilities and flexibilities upon application. Biology has a unique advantage in dynamic response; thus, how to integrate biological and non‐biological components into responsive “smart” materials is the focus. Most directional modifications at the molecular level can be achieved via synthetic biology techniques; however, beyond the cellular level, the collective behavior of the cell group and the unknown properties generated when the cells are in contact with inorganic materials are difficult to control because of factors such as quantity, environment, and state. This difficulty is particularly evident in the gap between proof‐of‐concept hybrid materials (including simple mixtures of cells and hydrogels) and large‐batch products.^[^ ^245^ ^]^

Third, the design‐build‐test‐learn (DBTL) cycle must be accelerated to discover and develop advanced biomaterials. The traditional research and development of biomaterials comprises a design‐build‐test, which has a long cycle and is easy to repeat. The rapid development of machine learning and its application in materials engineering offers the opportunity to predict the behaviors of biomolecules via a large amount of training, shorten the experimental cycle, and realize the leap in performance.^[^ ^246^ ^]^ Nevertheless, this combination is less effective. Quickly establishing this cycle can help drive the development of advanced biomaterials.

#### 6.1.2 Challenges in System Integration

Integration capabilities, one of the most prominent features, of biomimetic wearable sensing systems also presents several key challenges. First, the size and weight limitations of the devices impose requirements on integration capability. With the increasing demand for device functionality and emergence of (semi)implantable wearable devices, integrating more sensors and electronic components into limited or even extremely small spaces while maintaining device comfort and wearability has become a significant challenge. Second, for multisensory systems, the fusion and integration of data from different sensors pose a complex problem. Owing to differences between sensor types and acquisition methods, the accurate integration and processing of data from different sensors is a challenging task that needs to be addressed.^[^ ^196c^ ^]^ Moreover, energy supply is another key challenge for integration capability. Traditional battery‐powered solutions demonstrate limitations in terms of battery life, volume, and charging frequency. Therefore, the development of new energy supply technologies, such as energy harvesting and self‐powered materials, is an important direction for improving the integration capabilities of biomimetic wearable sensing systems.

#### 6.1.3 Challenges in System Stability

Stability is a critical challenge in the field of biomimetic wearable sensing systems. Sensing systems need to be in contact with the external environments such as humidity, temperature, and chemical substances, which can affect the materials and structures, thereby impacting the stability of the system performance. For example, changes in humidity can cause materials to absorb or dry, thereby altering the electrical or mechanical properties of the systems.^[^ ^72^ ^]^ Flexible sensors, as important technological tools, are widely applied in biomimetic wearable systems. However, due to the unique nature of flexible sensors, their stabilities have always been one of the main limiting factors for their applications. Flexible sensors typically use organic materials as the sensitive layer; nevertheless, because of their relatively unstable molecular structures, these materials may undergo aging and corrosion during long‐term usage, thereby affecting the stabilities of the systems. However, flexible sensors need to adapt to different strain environments including bending, stretching, and twisting. Nevertheless, these mechanical stresses can lead to changes in the system performance or even damage the system, thus affecting stabilities of the systems.

#### 6.1.4 Challenges in Achieving Advanced Transmission Capabilities

To attain advanced sensing capabilities, biomimetic sensors should exhibit target recognition accuracies comparable to those of human senses, thereby unlocking their full potentials. However, current biomimetic sensing technology still needs to achieve this target precision, thereby presenting a formidable challenge that must be overcome.^[^ ^24^ ^]^ To overcome this challenge, researchers have proposed various promising technologies, such as nanosensors, machine learning, and robotics, to alleviate the inherent limitations of artificial sensing systems. These technologies have demonstrated the potentials to improve the performances of sensing systems by compensating for an insufficient number of effective receptors, enhancing signal processing efficiency, and improving adaptability to different environments and situations. Via continuous development and optimization, these technologies are expected to substantially improve the accuracies and recognition abilities of artificial sensing systems and even surpass the capabilities of human sensory systems. Therefore, although the most advanced bionic sensing technology currently fails to achieve target recognition accuracy comparable to those of human sensory systems, we believe that future research and technological innovation will help overcome this daunting challenge.

#### 6.1.5 Challenges in Introducing Machine Learning Algorithms

Presently, the challenge in applying machine learning to biomimetic wearable sensors is handling and interpreting complex and dynamically changing sensor data. Specifically, this requires overcoming difficulties in sensor signal noise, processor performance limitations, data acquisition and processing complexity, and selecting and optimizing appropriate machine learning algorithms and models to maximize sensor performance and accuracy.^[^ ^205^ ^]^ Additionally, addressing data privacy and security issues is necessary to ensure the integrity and confidentiality of sensor data.

#### 6.1.6 Challenges in Introducing Synthetic Biology

Although synthetic biology demonstrates significant potential and promising applications, challenges remain in the targeted modification of non‐model organisms due to the inherent problems of bioengineering.^[^ ^245^ ^]^ Traditional gene modification, transformation, and screening techniques require considerable time and resources and are easily affected by environmental factors. Thus, more efficient and accurate techniques are needed to address this problem. To overcome these challenges, innovative solutions based on synthetic biology must be constantly explored. We believe that by developing more efficient and accurate biological designs and optimization algorithms using artificial intelligence and other technologies, the challenges of targeted modification can be addressed. Additionally, new high‐throughput screening techniques and in vitro synthesis technologies should be explored to rapidly screen and produce synthetic organisms. Moreover, in‐depth research and discussion on biological safety and ethical issues are needed to ensure the sustainability and safety of synthetic biology.

### 6.2 Outlook

As an emerging technology, biomimetic wearable sensors can monitor and analyze various physiological and environmental parameters of the human body, thereby providing people with more accurate, personalized, and convenient health and safety services. In the future, with the continuous development of technology and expansion of application scenarios, biomimetic wearable sensors are expected to overcome the limitations of traditional sensors in terms of accuracy, stability, and real‐time performance, realizing more precise and personalized data acquisition and analysis and offering more convenience and security guarantees to people's lives and work.

With regard to the development of the system, material innovation and system accuracy should be the focus of our efforts. In terms of material innovation, 4D printing can help shorten the cycle of material testing and modification, promote the fabrication of advanced materials, and create “smart” materials with autonomous structural deformation capabilities, rendering wearable devices more lightweight, flexible, and breathable and improving user experience. In terms of system accuracy, biomimetic neural network technology can be incorporated into biomimetic wearable sensors to endow these sensors with the abilities to mimic the structures and functions of human brain neurons, thereby improving their accuracies and intelligence levels. Additionally, gradually realizing closed‐loop control of the perception–decision–execution of biomimetic wearable sensors using the advantages of biomimetic neural networks is also one of the future development directions in this field.

Moreover, the enhancement of integration capability and stability is equally crucial for the advancement of the system. In terms of integration capability, progress in micro/nanotechnology and flexible electronics provides new opportunities for the integration of device components. The development of artificial intelligence and machine learning has demonstrated advantages in multimodule data processing. Novel wireless energy transfer and self‐powered materials hold promises for offering more convenient and durable solutions for the energy supply of devices. Utilization of cutting‐edge technologies, such as nanomaterials and smart materials, has the potential to further improve the stabilities of biomimetic wearable sensors. Furthermore, further research on the mechanisms of interaction between sensors and the external environment and impact of human physiological characteristics on sensor stability can contribute to better addressing of stability issues.

With the continuous advancement of technologies including bioelectronics, chips, and artificial intelligence, biomimetic wearable sensors are becoming essential components of an intelligent society. In terms of medical applications, biomimetic wearable sensors are progressively realizing personalized medical care by collecting physiological data of users and providing doctors with more accurate diagnoses and treatment plans. In terms of health management, biomimetic wearable sensors are gradually realizing full‐life‐cycle health management, offering personalized health suggestions and warnings via intelligent data analysis and feedback. In the field of security, biomimetic wearable sensors are expected to progressively realize applications such as identity verification and access control management.

In summary, in the future, biomimetic wearable sensors will demonstrate applications in multiple fields, including medical care, health, sports, and security, and their application scenarios will continue to expand. There is huge development space in materials, systems, and applications, and biomimetic wearable sensors are becoming an important pillar of an intelligent society in the future, offering more convenience and efficiency to people's lives and work.

## Conflict of Interest

The authors declare no conflict of interest.
